# An unusual activity of mycobacterial MutT1 Nudix hydrolase domain as a protein phosphatase regulates nucleoside diphosphate kinase function

**DOI:** 10.1128/jb.00314-24

**Published:** 2024-12-11

**Authors:** Elhassan Ali Fathi Emam, Koyel Roy, Devendra Pratap Singh, Deepak K. Saini, Umesh Varshney

**Affiliations:** 1 Department of Microbiology and Cell Biology, Indian Institute of Science 200195 , Bangalore, India; 2 Developmental Biology and Genetics, Indian Institute of Science 196383 , Bangalore, India; 3 Jawaharlal Nehru Centre for Advanced Scientific Research 29130 , Bangalore, India; University of Notre Dame, Notre Dame, Indiana, USA

**Keywords:** *Mtb*MutT1, *Msm*MutT1, *Msm*MutT2, *Eco*MutT, *Mtb*NDK, *Msm*NDK, *Eco*NDK, *Eco-Msm*MutT1, 8-oxo-(d)GTP, 8-oxo-(d)GDP, Nudix box, mutation frequency

## Abstract

MutT proteins are Nudix hydrolases characterized by the presence of a Nudix box, GX5EX7REUXEEXGU, where U is a bulky hydrophobic residue and X is any residue. Major MutT proteins hydrolyze 8-oxo-(d)GTP (8-oxo-GTP or 8-oxo-dGTP) to the corresponding 8-oxo-(d)GMP, preventing their incorporation into nucleic acids. Mycobacterial MutT1 comprises an N-terminal domain (NTD) harboring the Nudix box motif, and a C-terminal domain (CTD) harboring the RHG histidine phosphatase motif. Interestingly, unlike other MutTs, the MutT1 hydrolyses the mutagenic 8-oxo-(d)GTP to the corresponding 8-oxo-(d)GDP. Nucleoside diphosphate kinase (NDK), a conserved protein, carries out reversible conversion of (d)NDPs to (d)NTPs through phospho-NDK (NDK-*Pi*) intermediate. Recently, we showed that NDK-*Pi* converts 8-oxo-dGDP to 8-oxo-dGTP and escalates A to C mutations in a MutT-deficient *Escherichia coli*. We now show that both *Mycobacterium tuberculosis* MutT1 and *Mycobacterium smegmatis* MutT1, through their NTD (Nudix hydrolase motifs) function as protein phosphatase to regulate the levels of NDK-*Pi* and prevent it from catalyzing conversion of (d)NDPs to (d)NTPs (including conversion of 8-oxo-dGDP to 8-oxo-dGTP). To corroborate this function, we show that *Msm*MutT1 decreases A to C mutations in *E. coli* under the conditions of *Eco*NDK overexpression.

**IMPORTANCE:**

MutT proteins, having a Nudix box domain, hydrolyze the mutagenic 8-oxo-dGTP to 8-oxo-dGMP. However, mycobacterial MutT (MutT1) comprises an N-terminal domain (NTD) harboring a Nudix box, and a C-terminal domain (CTD) harboring an RHG histidine phosphatase. Unlike other MutTs, mycobacterial MutT1 hydrolyses 8-oxo-dGTP to 8-oxo-dGDP. Nucleoside diphosphate kinase (NDK), a conserved protein, converts 8-oxo-dGDP to 8-oxo-dGTP through phospho-NDK (NDK-*Pi*) intermediate and escalates A to C mutations. Here, we show that the mycobacterial MutT1 is unprecedented in that its NTD (Nudix box), functions as protein phosphatase to regulate NDK-*Pi* levels and prevents it from converting dNDPs to dNTPs (including 8-oxo-dGDP to 8-oxo-dGTP conversion). In addition, mycobacterial MutT1 decreases A to C mutations in *Escherichia coli* under the conditions of NDK overexpression.

## INTRODUCTION

*Mycobacterium tuberculosis*, a pathogenic bacterium, homes the host macrophages, where it encounters high levels of reactive oxygen species (ROS) and reactive nitrogen intermediates (RNI) generated as part of the host’s innate immune response (1, 2). ROS and RNI can damage nucleotide bases in nucleic acids and the free nucleotide pool. Due to its low redox potential, guanine (G) is highly susceptible to oxidative damage. Oxidation of (d)GDP (GDP/dGDP) or (d)GTP (GTP/dGTP) converts them to 8-oxo-(d)GDP or 8-oxo-(d)GTP, respectively (3–5). As 8-oxo-dGTP can pair with A, its misincorporation into the genome may result in AT to CG transversion mutations (6, 7).

The 8-oxo-(d)GDP can also be converted to 8-oxo-dGTP by nucleoside diphosphate kinase (NDK) (8). NDK is an evolutionarily conserved enzyme responsible for multiple cellular functions. The pathogens like *M. tuberculosis*, secrete NDK (*Mtb*NDK) as an effector protein that regulates diverse host cellular processes, including phagocytosis, apoptosis/necrosis, and inflammatory response (9, 10). *Mtb*NDK forms a stable hexamer (11). A major role of NDK is to maintain (homeostasis) the intracellular nucleoside diphosphate and nucleoside triphosphate pools (12–14). The catalysis by NDK involves autophosphorylation of a conserved His in it by the γ-phosphate of nucleoside triphosphates. The phosphate from His-*Pi* is then transferred to nucleoside diphosphates through a ping-pong mechanism, converting them into nucleoside triphosphates (12, 14). Recently, we observed that NDK catalyzes the formation of 8-oxo-dGTP from 8-oxo-dGDP at a rate 2–3 times higher than its activity in converting 8-oxo-dGTP into 8-oxo-dGDP (8). However, no significant differences were observed in the rates of reversible conversions between GTP and GDP. Importantly, overexpression of NDK in *Escherichia coli* Δ*mutT* increases A to C mutations (8).

MutT proteins belong to the Nudix hydrolase family, which possesses a Nudix hydrolase motif (GX5EX7REUXEEXGU). In this motif, U indicates a bulky hydrophobic residue, whereas X represents any residue (15, 16). *E. coli* MutT hydrolyses 8-oxo-(d)GTP and 8-oxo-(d)GDP into 8-oxo-(d)GMP (17). In mycobacteria*,* four of the Nudix hydrolases (MutT1, MutT2, MutT3, and MutT4) were identified as orthologs of *E. coli* MutT. Of these, MutT1 hydrolyzes 8-oxo-(d)GTP into 8-oxo-(d)GDP. Hydrolysis of 8-oxo-dGTP to 8-oxo-dGDP also prevents its misincorporation into the genome (18, 19). Mycobacterial MutT1 complements *E. coli* CC101 (Δ*mutT*) strain and decreases A to C mutations (19). MutT2 functions majorly as an efficient dCTPase (20). It has been reported that the mycobacterial MutT3 (RenU) is required for the pathogen to survive under the environment of oxidative stress inside macrophages (21). The *in vitro* experiments revealed that MutT4 exhibits greater efficacy in the hydrolysis of dATP. Its deficiency in the *mutT4* mutant strain results in a rise in the ratio of A to G (or T to C) mutations under oxidative stress (22, 23). Unlike *E. coli* MutT or the mycobacterial MutT2, MutT3, and MutT4, the mycobacterial MutT1 is unusual in consisting of an N-terminal domain (NTD) harboring a Nudix hydrolase motif, and a C-terminus domain (CTD) harboring an RHG histidine phosphatase motif of unknown activity (18, 24).

Although NDK is involved in diverse biological processes (12) and is autophosphorylated (to NDK-*Pi*) on a conserved His, no studies are available that have explored its possible regulation by dephosphorylation of its phospho-His by any of the cellular proteins. Because of the cross-talk between MutT and NDK in *E. coli*, we wondered if NDK could be a substrate for dephosphorylation by mycobacterial MutT1.

Our biochemical and *in vivo* studies, designed to investigate the possible roles of the NTD and CTD of MutT1 in regulation of NDK, reveal that NDK-*Pi* is a substrate for dephosphorylation by MutT1. While the dephosphorylation of NDK-*Pi* is catalyzed by the NTD of MutT1, we show that the CTD of MutT1 is important in supporting the phosphatase activity of the NTD. Finally, we show how MutT1 regulates the activity of NDK both *in vitro* and *in vivo*.

## RESULTS

### Mycobacterial MutT1 exhibits phosphatase activity against NDK-*Pi*

**Fig 1:**
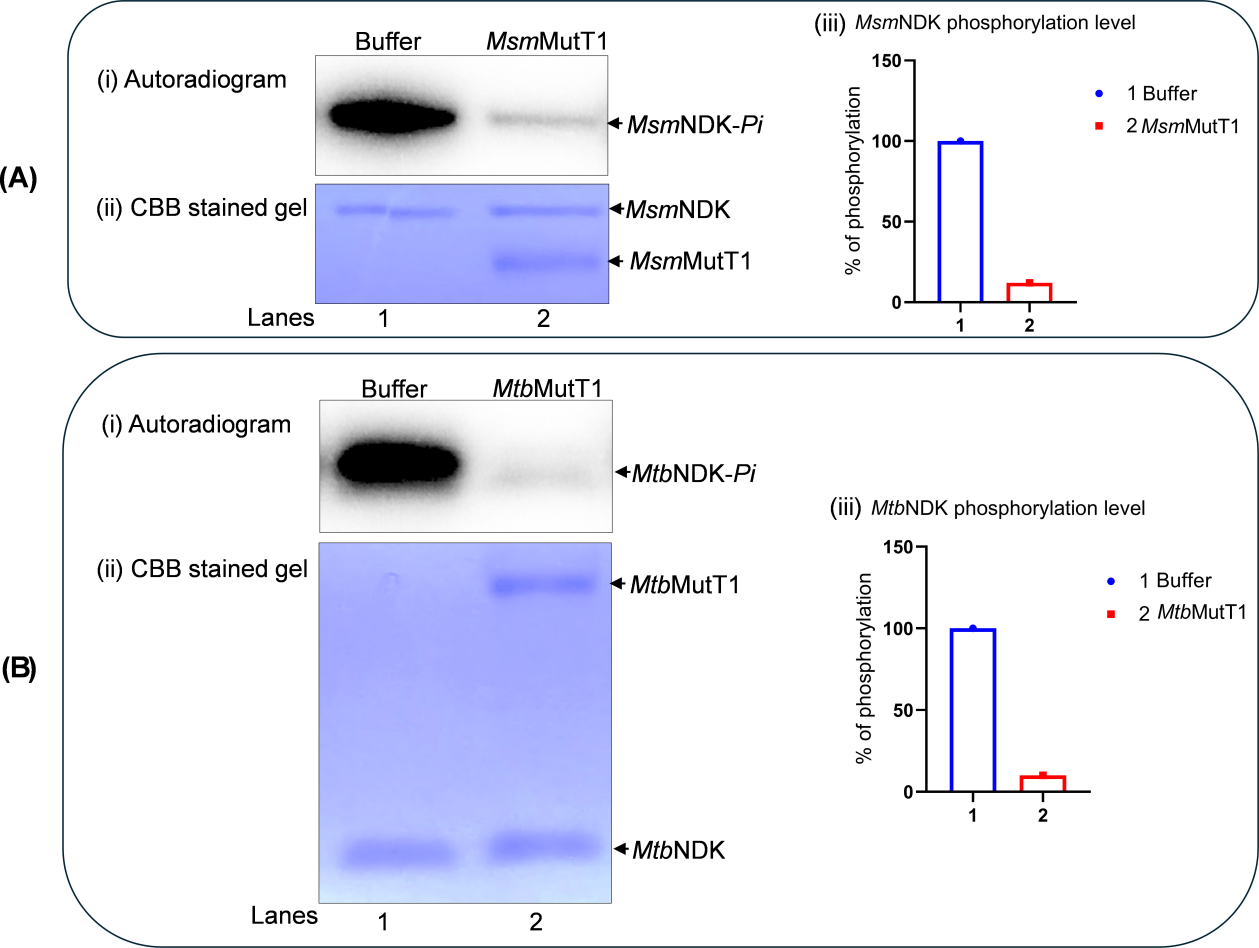
Dephosphorylation of NDK-*Pi* with mycobacterial MutT1. (**A**) Dephosphorylation of *Msm*NDK-*Pi* by *Msm*MutT1. *Msm*NDK was autophosphorylated in the presence of γ-^32^P-ATP to obtain *Msm*NDK-*Pi*. The *Msm*NDK-*Pi* (1 µg) was then incubated with either buffer alone (lane 1) or 1 µg *Msm*MutT1 (lane 2) at 30°C for 1 h, mixed with 5 µL SDS-PAGE sample buffer (without heating) and analyzed on 12% SDS-PAGE (because the phospho-His is labile to heat, the samples were not heated). Gels were subjected to phosphor imaging, fixed, and stained with Coomassie brilliant blue (CBB). Panels (i) and (ii) represent autoradiogram, and CBB stained gel, respectively, (iii) bar graph of quantification of the NDK phosphorylation levels. (**B**) Dephosphorylation of *Mtb*NDK-*Pi* by *Mtb*MutT1. The *Mtb*NDK-*Pi* (1 µg) obtained as in (**A**), was incubated with buffer alone (lane 1) or *Mtb*MutT1 (1 µg) (lane 2). The reactions were processed as in (**A**) above. Panels: (i) autoradiogram, (ii) CBB stained gel, and (iii) bar graph of quantification of the NDK phosphorylation level.

To test if autophosphorylated NDK (NDK-*Pi*) is a substrate for MutT1 phosphatase activity, we purified *Msm*NDK, *Mtb*NDK, *Msm*MutT1, and *Mtb*MutT1 (Fig. S1A). As reported earlier (18, 19), in this study too we observed that *Msm*MutT1 and *Mtb*MutT1 migrate as doublets on SDS-PAGE. The assays for the phosphatase activity using *p*NPP as a generic substrate revealed that both the MutT1 proteins were active (Fig. S1B, compare bars 2 and 3 with bar 1 control). Incubation of *Msm*NDK-*Pi* with *Msm*MutT1 showed that the MutT1 resulted in dephosphorylation of *Msm*NDK-*Pi* (Fig. 1A, *panel i*, compare lane 2 with lane 1). SDS-PAGE analysis of the same gel (Fig. 1A, *panel ii*), showed that equal amounts of *Msm*NDK were present in both the lanes and that its dephosphorylation occurred only in the sample containing *Msm*MutT1. Quantification showed that dephosphorylation of *Msm*NDK-*Pi* by *Msm*MutT1 was ~90% (Fig. 1A, *panel iii*). Identical observations were made in the assays with *Mtb* proteins (*Mtb*NDK and *Mtb*MutT1) (Fig. 1B), revealing that MutT1 has a phosphatase activity against NDK-*Pi*.

### Generation of single-site mutations in NTD and CTD of MutT1 proteins

Mycobacterial MutT1 comprises two domains, NTD having Nudix hydrolase motif, and CTD having RHG histidine phosphatase motif. To better understand the role of MutT1 in dephosphorylation of NDK-*Pi*, we generated single-site mutations of E81A (in NTD) or H170A (in CTD) in *Msm*MutT1 (Fig. S2A). The E81 and H170 are critical residues in the Nudix box and the RHG His-phosphatase motifs, respectively (23, 25, 26). The assays using the mutant proteins (Fig. S2B) showed that the E81A mutation in NTD rendered MutT1 inactive in dephosphorylation of *p*NPP (Fig. S2C, compare bar 3 with bars 1 and 2). However, the H170A mutation in CTD had no impact on the dephosphorylation of *p*NPP (Fig. S2C, compare bar 4 with bars 1 and 2). The equivalent mutations in *Mtb*MutT1 corresponded to E69A in the Nudix hydrolase motif in NTD, and H161A in the RHG motif in CTD (Fig. S3A). Consistent with the *Msm*MutT1 mutants, *Mtb*MutT1 E69A but not the *Mtb*MutT1 H161A lost its dephosphorylation activity on *p*NPP (Fig. S3B and C).

### Nudix hydrolase motif is the source of protein phosphatase activity on NDK-*Pi*

**Fig 2:**
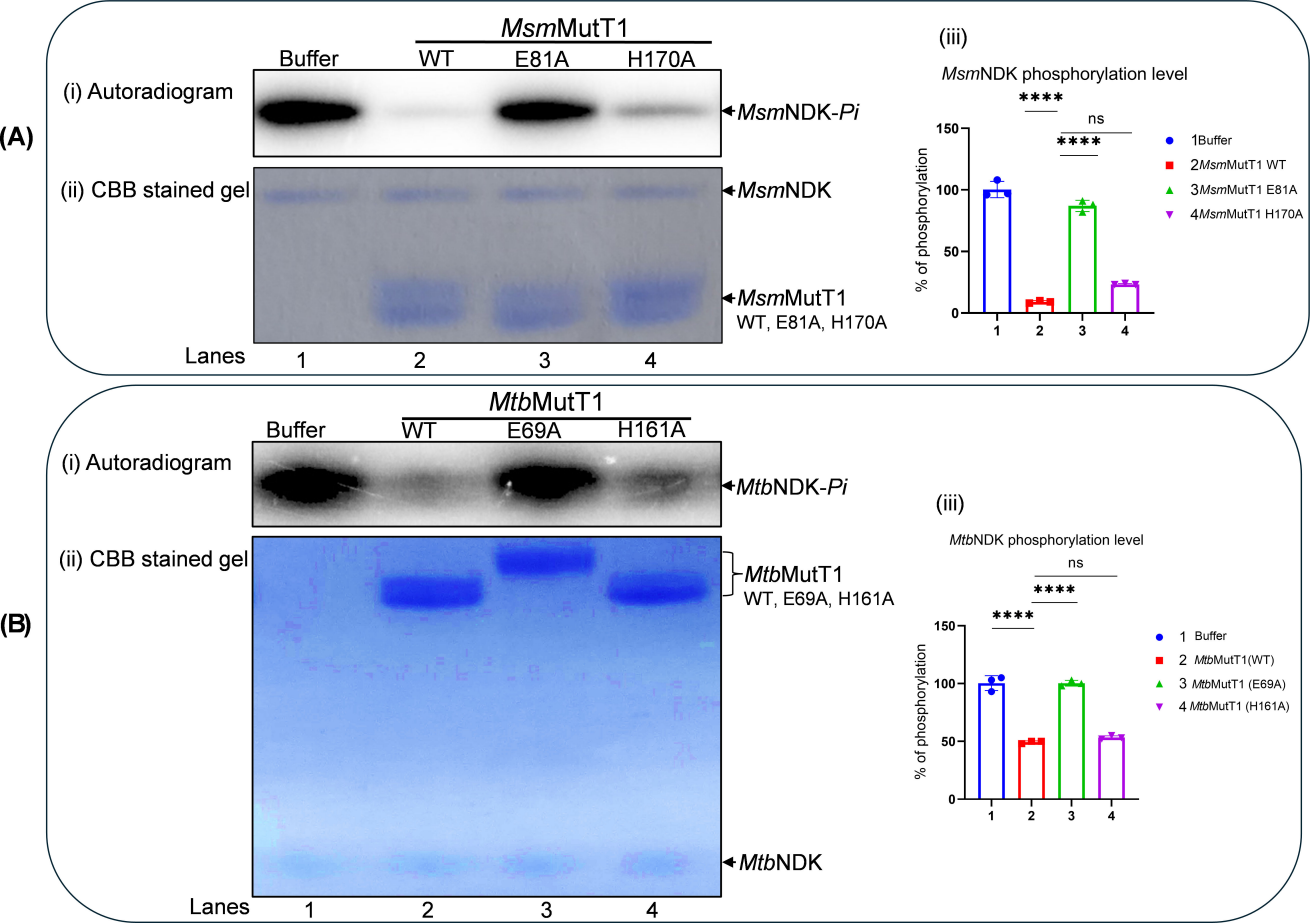
Dephosphorylation of NDK-*Pi* with MutT1 mutants. (**A**) Dephosphorylation of *Msm*NDK-*Pi* by *Msm*MutT1 (WT, E81A, and H170A). The autophosphorylated *Msm*NDK-*Pi* (1 µg) was incubated with buffer alone (lane 1) or 2 µg *Msm*MutT1 (lane 2), or 2 µg *Msm*MutT1 E81A (lane 3), or 2 µg *Msm*MutT1 (H170A) (lane 4). Panels (i) and (ii) represent autoradiogram and CBB stained gel, respectively. It may be noted that because the samples are not heated after adding sample dye, *Msm*NDK (hexamer) migrates much slower than its expected monomeric molecular weight (also refer to Fig. **S7**). (iii) Bar graph of phosphorylation levels of *Msm*NDK incubated with buffer, *Msm*MutT1, *Msm*MutT1 E81A or *Msm*MutT1 H170A (bars 1, 2, 3, and 4, respectively). Bars represent mean ± SD for *n* = 3. *P* values, ∗∗ <0.01; ∗∗∗ <0.001; ∗∗∗∗ <0.0001 indicate significant differences between samples; “ns” represent not significant. One-way analysis of variance (ANOVA) method was used to calculate the *P* value. Three technical replicates were used. (**B**) Dephosphorylation of *Mtb*NDK-*Pi* by *Mtb*MutT1 (WT, E69A, and H161A). The *Mtb*NDK-*Pi* (1 µg) was incubated with buffer alone (lane 1), or 2 µg *Mtb*MutT1 (lane 2), or 2 µg *Mtb*MutT1 E69A (lane 3), or 2 µg *Mtb*MutT1 H161A (lane 4). Panels (i) and (ii) represent autoradiogram, and CBB stained gel, respectively. (iii) Bar graph of the phosphorylation levels of *Mtb*NDK incubated with buffer, *Mtb*MutT1 *Mtb*MutT1 E69A or *Mtb*MutT1 H161A (bars 1, 2, 3, and 4, respectively). Bars represent mean ± SD for *n* = 3. *P* values, ∗∗ <0.01; ∗∗∗ <0.001; ∗∗∗∗ <0.0001 indicate significant differences between samples; “ns” represent not significant. The one-way ANOVA method was used to calculate the *P* value. Three technical replicates were used.

To check which of the domains of MutT1 exhibited the phosphatase activity on NDK-*Pi*, *Msm*NDK-*Pi* was treated with *Msm*MutT1, *Msm*MutT1(E81A), or *Msm*MutT1(H170A). The results in Fig. 2A show that dephosphorylation of *Msm*NDK-*Pi* occurred upon treatment with *Msm*MutT1 and *Msm*MutT1 H170A (Fig. 2A, *panel i*, lanes 2 and 4; panel *iii*, bars 2 and 4), respectively. No significant dephosphorylation was detected when the *Msm*NDK-*Pi* was incubated with *Msm*MutT1 E81A (Fig. 2A, *panel i*, compare lane 3 with the buffer alone lane 1; panel *iii*, compare bar 3 with bar 1). Similar results were obtained when *Mtb*NDK-*Pi* was treated with *Mtb*MutT1, *Mtb*MutT1 E69A, or *Mtb*MutT1 H161A (Fig. 2B). The SDS-PAGE analysis (Fig. 2A and B, *panels ii*) shows that equal amounts of NDK and MutT1 proteins were used. Taken together, these findings show that mutation in the NTD Nudix box (*Msm*MutT1 E81A and *Mtb*MutT1 E69A) resulted in the loss of their dephosphorylation activities on NDK-*Pi* suggesting that the Nudix hydrolase motif of the MutT1 proteins is responsible for dephosphorylation of NDK-*Pi. Msm*NDK and *Mtb*NDK share ~80% sequence similarity (Fig. S4A). Not unexpectedly, we observed efficient dephosphorylation of *Msm*NDK-*Pi* by both *Msm*MutT1 and *Mtb*MutT1 but not the NTD mutants (Fig. S4B). Likewise, *Mtb*NDK-*Pi* is dephosphorylated with either of the MutT1 but not the NTD mutants (Fig. S4C). These data show that MutT1 proteins act not only on their homologous NDKs but also on those from different mycobacterial species. Importantly, these observations further consolidate the role of MutT1 NTD in NDK-*Pi* dephosphorylation.

### MutT1 CTD supports the phosphatase activity of MutT1 NTD

**Fig 3:**
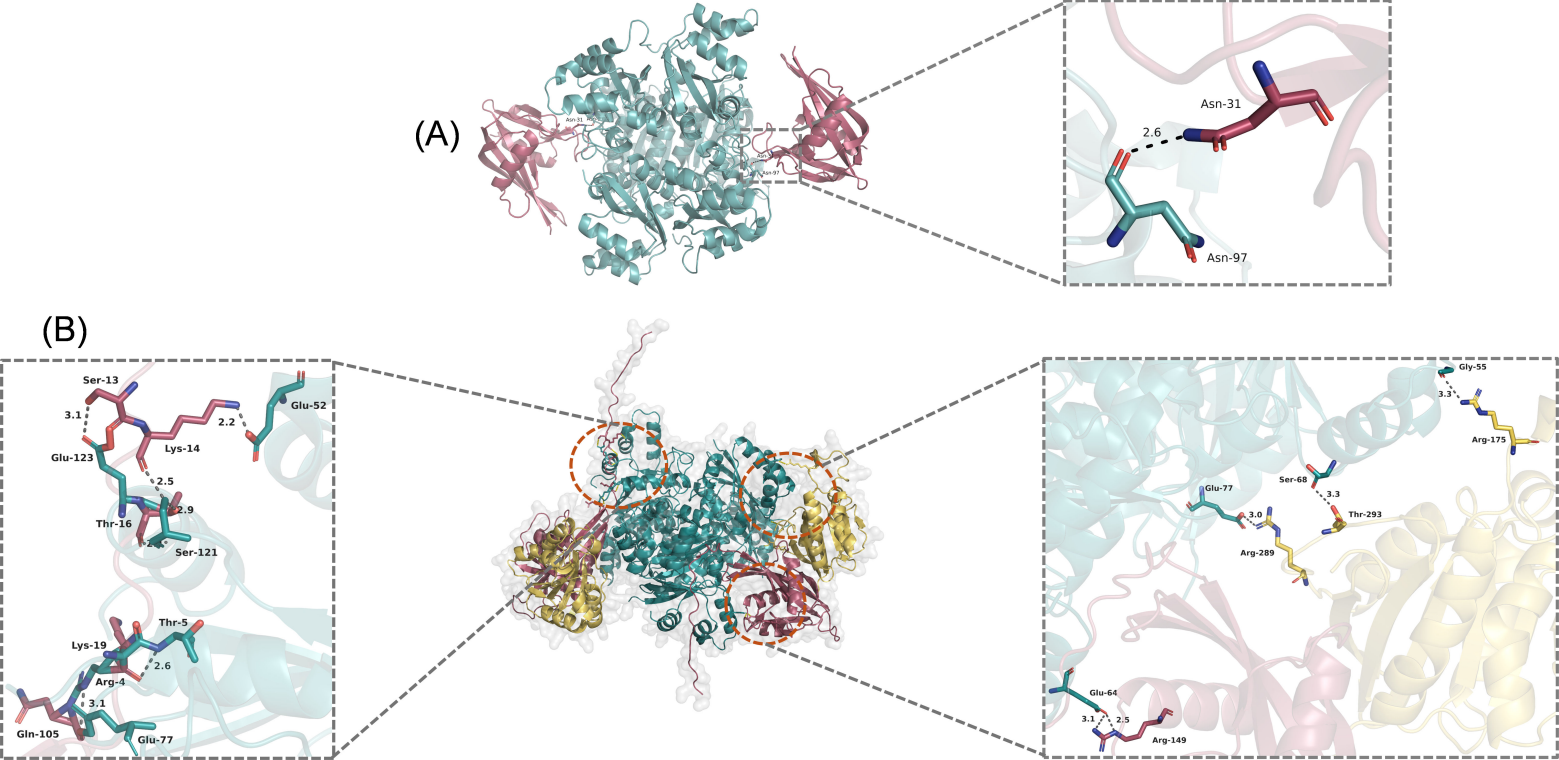
Protein-protein interaction prediction using AlphaFold colab. (**A**) The protein sequences of 2X *Eco*MutT (dark red) + 6X *Eco*NDK (cyan) were submitted to the AlphaFold colab server for interaction prediction (26). The results were analyzed using Edu PyMol software (29). We could detect a single interaction between Asn31 from *Eco*MutT (in *“red”*) with Asn97 from *Eco*NDK (cyan). (**B**) The sequences of 2X *Msm*MutT1 + 6X *Msm*NDK (in *“cyan”*) were submitted to the AlphaFold colab server for interaction prediction (26). The results were analyzed using Edu PyMol software (29). We could observe the interaction of *Msm*NDK with both *Msm*MutT1 domains, *Msm*MutT1 NTD (*red*) and *Msm*MutT1 CTD (*yellow*).

The observations (Fig. 2) that the Nudix hydrolase is responsible for dephosphorylation of NDK-*Pi* raises a question if other MutT proteins (possessing a single domain corresponding to the mycobacterial MutT1 NTD) would act on NDK-*Pi*. Thus, we purified *Eco*MutT, *Msm*MutT2, and *Eco*NDK (Fig. S5A). Both *Eco*MutT and *Msm*MutT2 showed strong phosphatase activity on *p*NPP (Fig. S5B). However, no dephosphorylation of *Eco*NDK-*Pi* was detected when treated with *Eco*MutT (Fig. S6A, *panels* i–iii). Similarly, no dephosphorylation of *Msm*NDK-*Pi* occurred with *Msm*MutT2 (Fig. S6B, *panels* i–iii). To better understand why *Eco*MutT does not dephosphorylate *Eco*NDK-*Pi*, we made use of the AlphaFold Colab protein-protein interaction tool (27, 28). We observed a single site interaction between *Eco*MutT and *Eco*NDK (Fig. 3A). While many interactions were observed between *Msm*NDK and *Msm*MutT1 (NTD) and (CTD) (Fig. 3B) indicating a possible requirement of MutT1 CTD in facilitating binding of NDK-*Pi* to MutT1 for dephosphorylation by MutT1 NTD.

**Fig 4:**
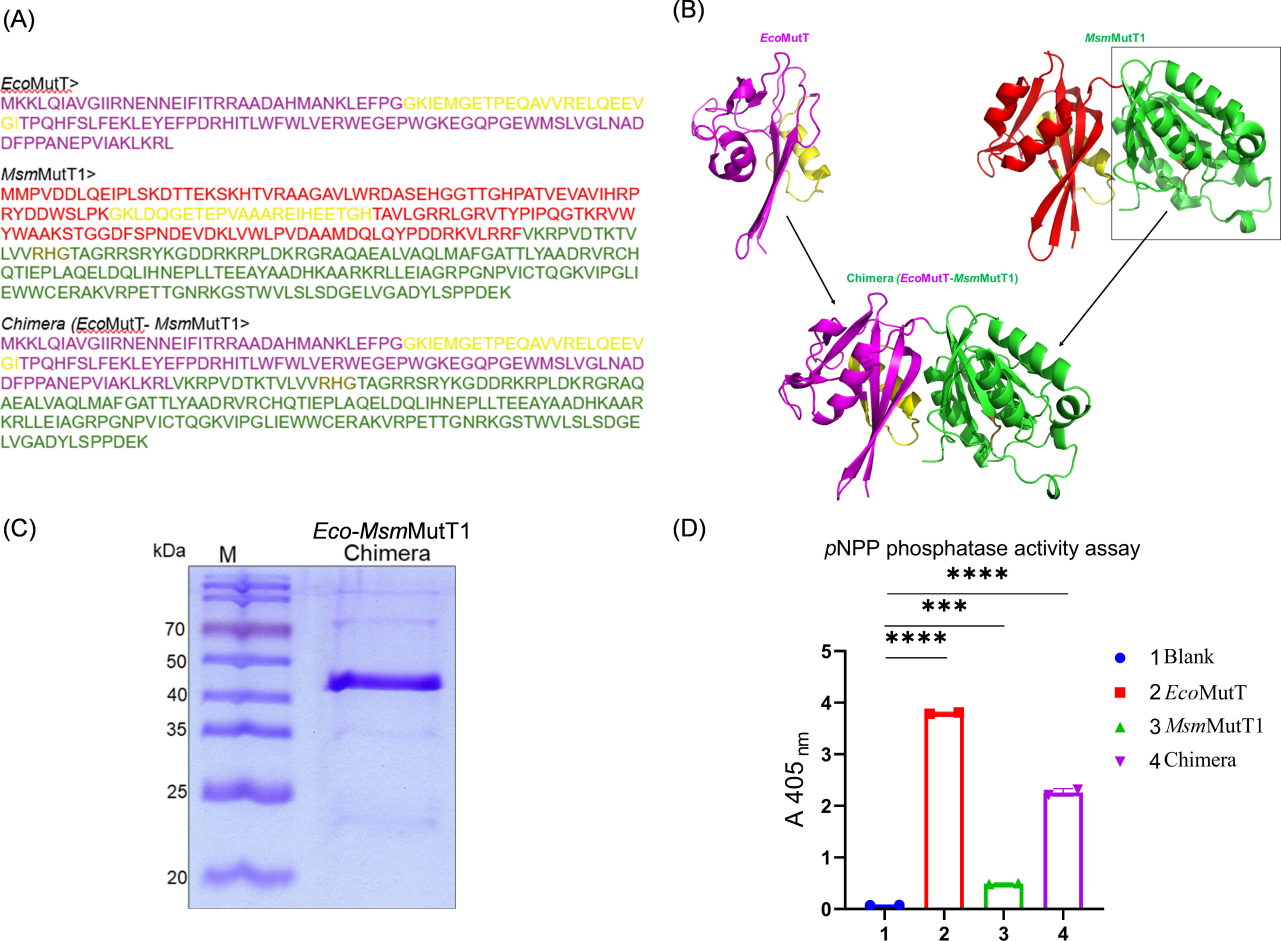
*Eco*MutT-*Msm*MutT1 (chimera) generation, purification, and its phosphatase activity. (**A**) Protein sequence of *Eco*MutT (magenta), *Msm*MutT1 (NTD red) and (CTD green), and *Eco-Msm*MutT1 (chimera). (**B**) Structure prediction for *Eco-Msm*MutT1 chimera. *Eco*MutT (*magenta*) PDB entry 1PUN (30), *Msm*MutT1 (NTD *red*) and (CTD *green*) (*Ms*MutT1; PDB entry 5GGB [21]) chimera structure prediction (27, 28). Sequences in yellow represent Nudix hydrolase motifs and in sand color represent the RHG motif. (**C**) Protein purification analysis on 15% SDS-PAGE gel showing the quality of the purified protein. *Eco-Msm*MutT1 chimera (~3 µg) was analyzed on 15% SDS-PAGE gel. The calculated molecular mass of *Eco-Msm*MutT1 chimera with His_6_ tag is ~37 kDa. (**D**) The graph demonstrates the general phosphatase activity of the purified proteins using *p*NPP as a substrate. The substrate was incubated with water (bar 1), 1 µg *Eco*MutT (bar 2), 1 µg *Msm*MutT1 (bar 3), or 1 µg *Eco-Msm*MutT1 chimera (bar 4). Bars represent mean ± SD for *n* = 3. *P* values, ∗ *P* < 0.05; ∗∗ *P* < 0.01; ∗∗∗ *P* < 0.001 indicate significant differences between samples; “ns” represent not significant. The one-way ANOVA method was used to calculate the *P* value.

**Fig 5:**
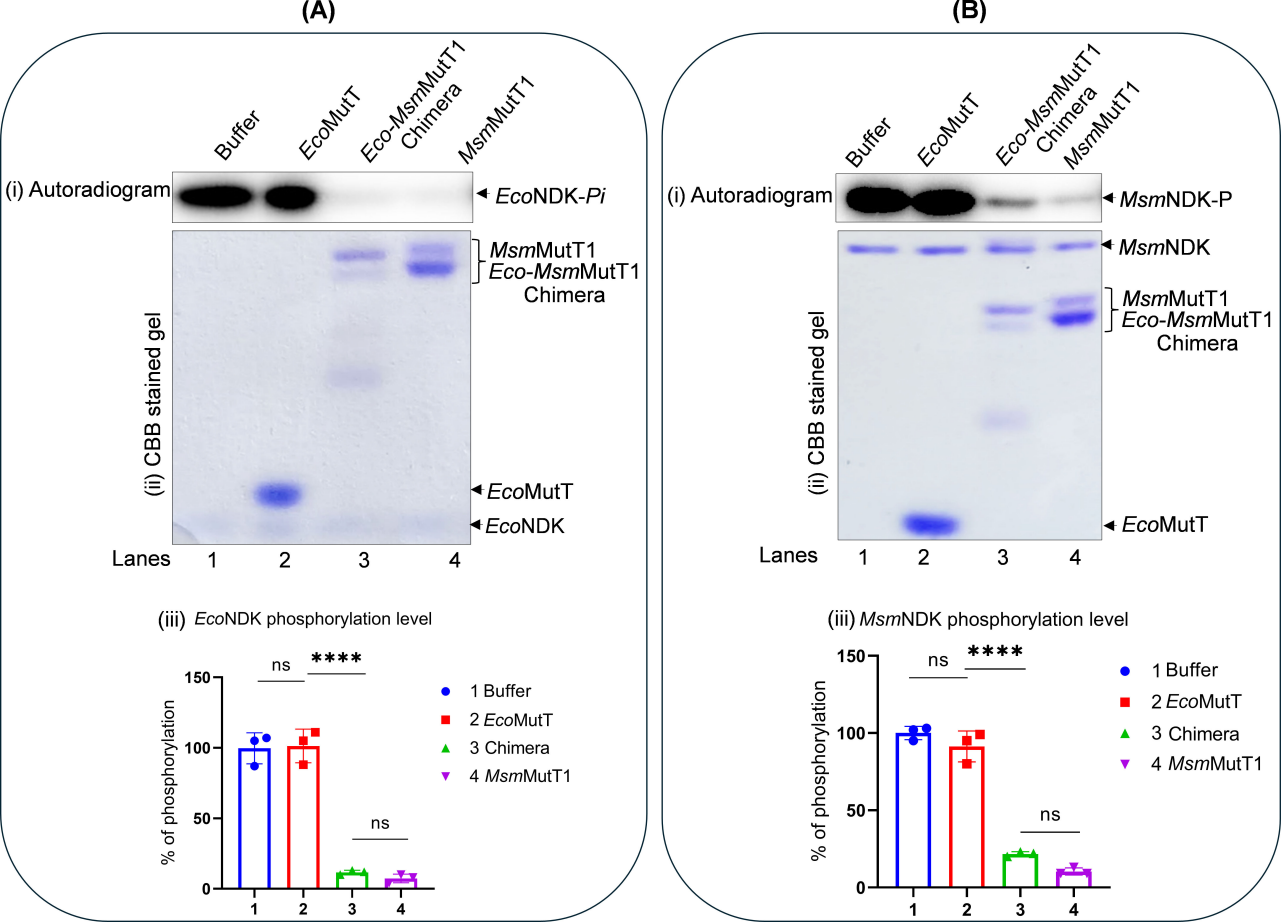
Dephosphorylation of NDK-*Pi* with *Eco-Msm*MutT1 chimera. (**A**) Dephosphorylation of *Eco*NDK-*Pi* by *Eco*MutT, *Eco-Msm*MutT1 chimera, and *Msm*MutT1. The autophosphorylated *Eco*NDK-*Pi* (0.5 µg) was incubated with buffer alone (lane 1), 2 µg *Eco*MutT (lane 2), 2 µg *Eco-Msm*MutT1 chimera (lane 3), or 2 µg *Msm*MutT1 (lane 4). Panels (i) and (ii) represent autoradiogram and CBB stained gel, respectively. (iii) Bar graph of quantification of the NDK phosphorylation levels. Bars represent mean ± SD for *n* = 3. *P* values, ∗∗ <0.01; ∗∗∗ <0.001; ∗∗∗∗ <0.0001 indicate significant differences between samples; “ns” represent not significant. The one-way ANOVA method was used to calculate the *P* value. Three technical replicates were used. (**B**) Dephosphorylation of *Msm*NDK-*Pi* by *Eco*MutT, *Eco-Msm*MutT1 chimera, and *Msm*MutT1. The *Msm*NDK-*Pi* (1 µg) was incubated with buffer alone (lane 1), 2 µg *Eco*MutT (lane 2), 2 µg *Eco-Msm*MutT1 chimera (lane 3), or 2 µg *Msm*MutT1 (lane 4). Panels (**I**) and (ii) represent autoradiogram and CBB stained gel. (iii) Bar graph of quantification of the NDK phosphorylation levels. Bars represent mean ± SD for *n* = 3. *P* values, ∗∗ <0.01; ∗∗∗ <0.001; ∗∗∗∗ <0.0001 indicate significant differences between samples; “ns” represent not significant. The one-way ANOVA method was used to calculate the *P* value. Three technical replicates were used. It may be noted that because the samples were not heated in the sample loading dye, *Msm*NDK (hexamer) migrates slower than its expected monomeric molecular weight (also refer to Fig. **S7**).

To test this prediction, we generated a chimeric protein where the NTD of *Msm*MutT1 was replaced with the *Eco*MutT sequence. The chimera was purified and assayed for its general phosphatase activity using *p*NPP (Fig. 4A through D). All the MutT proteins (*Eco*MutT, *Msm*MutT1, and *Eco-Msm*MutT1 chimera) showed the general phosphatase activity (Fig. 4D, compare bars 2–4 with 1). However, *Eco*MutT showed the best activity, and given that the Nudix hydrolase (NTD) conferred the phosphatase activity, the *Eco-Msm*MutT1, not unexpectedly, also showed a better activity compared with the *Msm*MutT1 (Fig. 4D, compare bars 3 and 4). Subsequently, *Eco*NDK-*Pi* was treated with *Eco*MutT, *Eco-Msm*MutT1 chimera, or *Msm*MutT1 (Fig. 5A). The results revealed a prominent decrease in the level of *Eco*NDK-*Pi* when incubated with *Eco-Msm*MutT1 chimera or *Msm*MutT1 (Fig. 5A *panel i*, lanes 3 and 4; *panel iii*, bars 3 and 4). As a control, no decrease in *Eco*NDK-*Pi* levels was detected upon its incubation with buffer alone or *Eco*MutT (Fig. 5A, *panel i*, lanes 1 and 2; *panel iii*, bars 1 and 2). Similar results were obtained when *Msm*NDK-*Pi* was used (Fig. 5B). The observations strongly suggest that *Msm*MutT1 CTD structurally contributes to MutT1 NTD phosphatase activity on NDK-*Pi*.

### MutT1 regulates NDK activity and its downstream function *in vitro*

**Fig 6:**
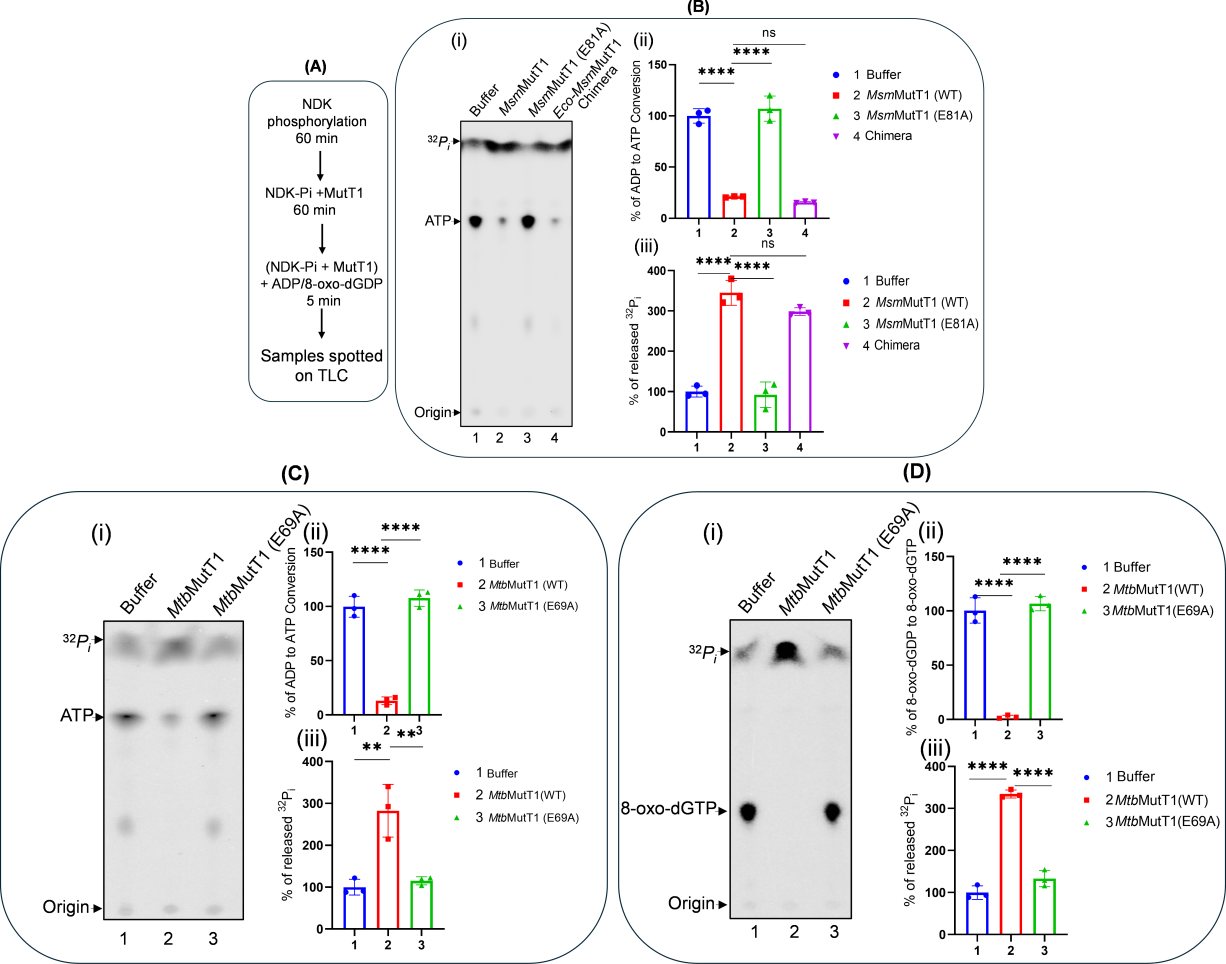
Regulation of NDK activity *in vitro*. (**A**) Schematic representation of the experimental design. (**B**) ADP to ATP conversion by *Msm*NDK. *Msm*NDK-*Pi* (1 µg) was incubated with buffer (lane 1), 2 µg *Msm*MutT1 (lane 2), 2 µg *Msm*MutT1 E81A (lane3), or 2 µg *Eco-Msm*MutT1 chimera (lane 4) at 30°C for 1 h followed by the addition of ADP as described in Materials and Methods. (ii) Bar graph showing % of ADP to ATP conversion by NDK. (iii) Bar graph showing % of released ^32^Pi. Bars represent mean ± SD for *n* = 3. *P* values, ∗∗ <0.01; ∗∗∗ <0.001; ∗∗∗∗ <0.0001 indicate significant differences between samples; “ns” represent not significant. The one-way ANOVA method was used to calculate the *P* value. Three technical replicates were used. (**C**) ADP to ATP conversion by *Mtb*NDK. The *Mtb*NDK-*Pi* (1 µg) was incubated with buffer (lane 1), 2 µg *Mtb*MutT1 (lane 2), or 2 µg *Mtb*MutT1 E69A (lane 3) at 30°C for 1 h followed by the addition of ADP as described in Materials and Methods. (ii) Bar graph showing % of ADP to ATP conversion by NDK. (iii) Bar graph showing % of released ^32^Pi. Bars represent mean ± SD for *n* = 3. *P* values, ∗∗ <0.01; ∗∗∗ <0.001; ∗∗∗∗ <0.0001 indicate significant differences between samples; “ns” represent not significant. The one-way ANOVA method was used to calculate the *P* value. Three technical replicates were used. (**D**) 8-oxo-dGDP to 8-oxo-dGTP conversion by *Mtb*NDK. The *Mtb*NDK-*Pi* (1 µg) was incubated with buffer (lane 1), 2 µg *Mtb*MutT1 (lane 2), or 2 µg *Mtb*MutT1 E69A (lane 3) for 1 h followed by the addition of 8-oxo-dGDP as described in Materials and Methods. (ii) Bar graph showing % of ADP to ATP conversion by NDK. (iii) Bar graph showing % of released ^32^Pi. Bars represent mean ± SD for *n* = 3. *P* values, ∗∗ <0.01; ∗∗∗ <0.001; ∗∗∗∗ <0.0001 indicate significant differences between samples; “ns” represent not significant. The one-way ANOVA method was used to calculate the *P* value. Three technical replicates were used.

NDK plays an important role in homeostasis of the cellular nucleotide pool crucial in maintaining genome integrity (8). It executes reversible conversions of NDPs to NTPs. To understand the effect of MutT1 on this function of NDK, *Msm*NDK-*Pi* was incubated with *Msm*MutT1, *Msm*MutT1 E81A, and the *Eco-Msm*MutT1 chimera, and followed further by incubation with ADP or 8-oxo-GDP (Fig. 6A). Observations in Fig. 6B show that *Msm*NDK-*Pi* converted ADP to ATP when it was pre-incubated with buffer alone or the Nudix hydrolase dead mutant *Msm*MutT1 E81A (Fig. 6B, *panel i*, lanes 1 and 3). However, the activity of *Msm*NDK-*Pi* in converting ADP to ATP was highly suppressed upon its pre-treatment with *Msm*MutT1 or the *Eco-Msm*MutT1 chimera (Fig. 6B, *panel i*, lanes 2 and 4, respectively). Quantifications of ADP to ATP conversion and Pi release (Fig. 6B, *panels ii* and *iii*) further confirm the observations. The same results were obtained when *Mtb*NDK-*Pi* was treated with the corresponding *Mtb*MutT1 proteins (Fig. 6C). We then asked if MutT1 restricts NDK-*Pi* from converting 8-oxo-dGDP to 8-oxo-dGTP. In this experiment also, *Mtb*MutT1 but not its E69A mutant, prevented the conversion of 8-oxo-dGDP to 8-oxo-dGTP (Fig. 6D). Radiolabeled ATP and NDK-*Pi* controls are shown in Fig. S8. These observations suggest that MutT1 regulates NDK activity in converting NDPs to NTPs.

### MutT1 regulates the NDK and affects its downstream activity *in vivo*

8-Oxo-dGTP misincorporation in DNA causes AT to CG transversions. *Msm*MutT1 hydrolyses 8-oxo-(d)GTP to 8-oxo-(d)GDP and prevents its misincorporation in DNA. However, overexpression of NDK in *E. coli* CC101 Δ*mutT* strain results in increased frequency of A to C mutations because of its role in the conversion of 8-oxo-dGDP to 8-oxo-dGTP (19). As MutT1 can dephosphorylate NDK-*Pi* and prevent it from converting 8-oxo-dGDP to 8-oxo-dGTP, we investigated the physiological relevance of this activity by utilizing *E. coli* CC101 Δ*mutT*Δ*ndk::kan*. The β-galactosidase (LacZ) gene in the CC101 strain is inactive because of an amber mutation (G to T) at the E461 codon (GAG to TAG) in the active site. For the strain to grow on lactose (as the sole carbon source) it requires a specific reversion mutation of A to C (or T to G) at this site. To investigate the effect of MutT1 on NDK, we carried out assays using the strains harboring pACDH*Eco*NDK.

**Fig 7:**
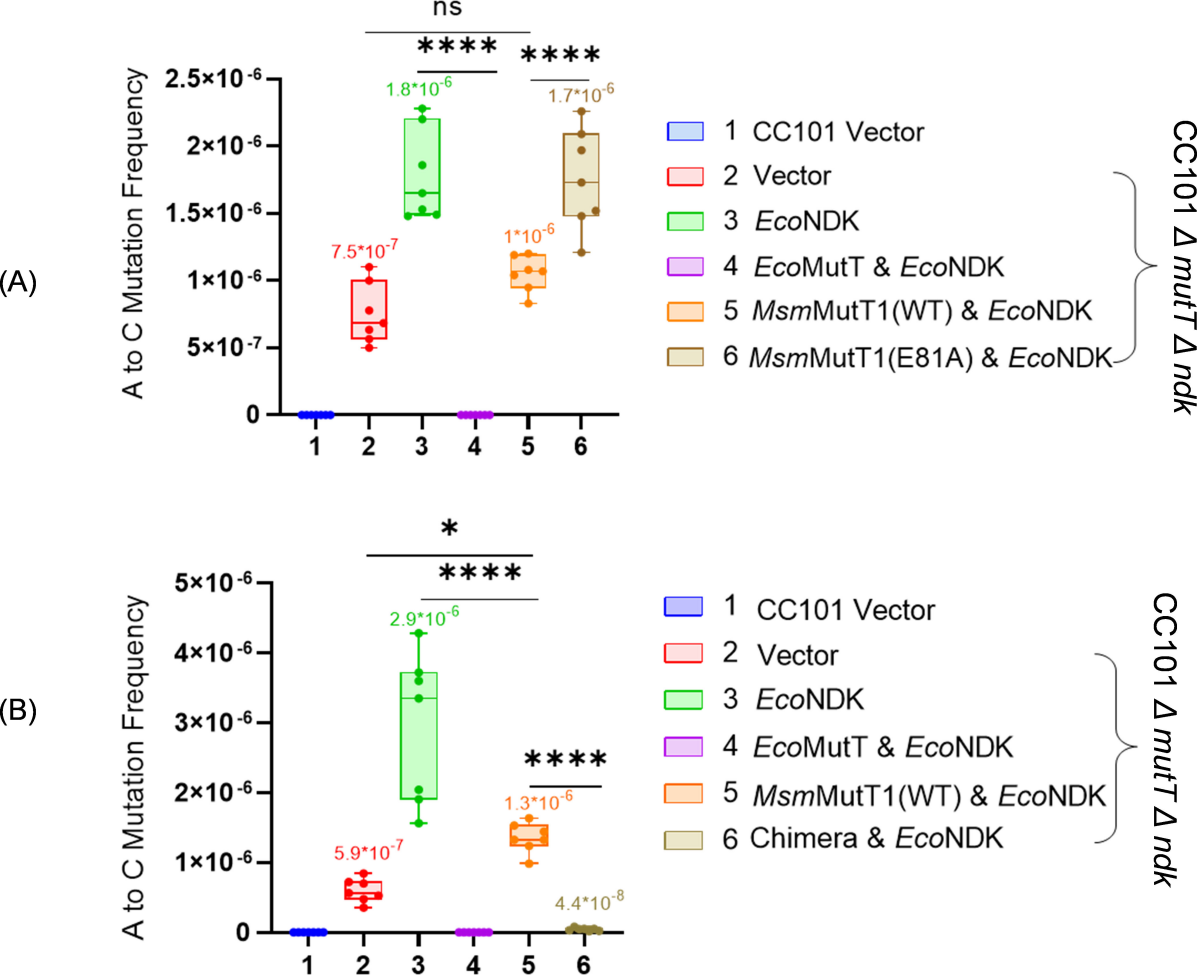
Regulation of NDK activity *in vivo*. Mutation frequency analysis of the Lac^+^ revertant with respect to A to C (or T to G) in *E. coli* CC101, CC101/pACDH*Eco*NDK, and its derivatives harboring (**A**) pBADHisB (empty vector), or its recombinants harboring *Eco*MutT, *Msm*MutT1 WT, or *Msm*MutT1 E81A. (**B**) Plasmids harboring *Eco*MutT, *Msm*MutT1 WT, or chimera. Reversion frequencies were calculated by dividing the number of colonies that appear on a minimal lactose plate by the number of colonies that appear on a minimal glucose plate. The data are represented as means ± SDs of 7 independent replicates. *P* values, ∗ *P* < 0.05; ∗∗ *P* < 0.01; ∗∗∗ *P* < 0.001 indicate significant differences between samples; “ns” represent not significant. The one-way ANOVA method was used to calculate the *P* value.

We transformed the CC101 Δ*mutT*Δ*ndk::kan*/pACDH*Eco*NDK strain with the plasmid-borne copies of *Eco*MutT, *Msm*MutT1, or *Msm*MutT1 (E81A), and determined the reversion frequencies (Fig. 7A). As expected, CC101Δ*mutT*Δ*ndk::kan*/pACDH*Eco*NDK showed a higher mutation frequency of 1.8 × 10^−6^ compared to the controls CC101/pACDH, CC101 Δ*mutT*Δ*ndk::kan*/pACDH*Eco*NDK/pBAD*Eco*MutT (undetectable mutation frequency) and CC101Δ*mutT*Δ*ndk::kan*/pACDH with mutation frequency of 7.5 × 10^−7^. The mutation frequency decreased to 1 × 10^−6^ when *CC101*Δ*mutT*Δ*ndk::kan/*pACDH*EcoNDK* was complemented with *Msm*MutT1. In comparison with *Msm*MutT1, complementation of *CC101*Δ*mutT*Δ*ndk::kan/*pACDH*EcoNDK* with *Msm*MutT1 (E81A), showed an increase in the mutation frequency (1.7 × 10^−6^). These observations are consistent with the *in vitro* observations of the MutT1-mediated regulation of NDK in converting 8-oxo-dGDP to 8-oxo-dGTP.

To investigate if *Eco-Msm*MutT1 rescued CC101Δ*mutT*Δ*ndk::kan/*pACDH*EcoNDK* strain*,* we transformed this strain with the plasmid-borne copies of *Eco*MutT, *Msm*MutT1, and the *Eco-Msm*MutT1 chimera, and determined the reversion frequencies (Fig. 7B). In this experiment too, CC101Δ*mutT*Δ*ndk::kan*/pACDH*Eco*NDK showed a higher mutation frequency of 2.9 × 10^−6^ compared to the controls CC101/pACDH, CC101 Δ*mutT*Δ*ndk::kan*/pACDH*Eco*NDK/pBAD*Eco*MutT (no mutation frequency was detected) and CC101Δ*mutT*Δ*ndk::kan/*pACDH with mutation frequency of 5.9 × 10^−7^. This frequency decreased to 1.3 × 10^−6^ when CC101Δ*mutT*Δ*ndk::kan/*pACDH*Eco*NDK was complemented with *Msm*MutT1. Complementation of CC101Δ*mutT*Δ*ndk::kan/*pACDH*Eco*NDK with *Eco-Msm*MutT1 chimera, showed an efficient decrease in the mutation frequency rate 4.4 × 10^−8^.

Thus, both the *in vitro* and *in vivo* observations (Fig. 6 and 7) suggest that the mycobacterial MutT1 regulates the function of NDK by dephosphorylating NDK-*Pi*.

## DISCUSSION

8-Oxo-dGTP which often results from the oxidation of dGTP, is a mutagenic nucleotide. The sources of oxidative agents can be the cellular metabolic activities or the external environments (31–34). 8-Oxo-dGTP can mis-pair with adenine and cause AT to CG transversion mutations (17, 35, 36). Tuberculosis, caused by *M. tuberculosis*, poses an alarming threat to global public health. It results in more deaths than those caused by diseases like COVID-19 and HIV/AIDS (1). *M. tuberculosis* encounters a high level of oxidative stress as part of the host’s innate immune response (2), and with ~65% GC-rich genome, it is highly prone to accumulate 8-oxo-dGTP in its DNA or the nucleotide pool (37). The role of MutT proteins is important in protection against the misincorporation of the 8-oxo-dGTP, and 8-oxo-GTP into nucleic acids (17–20, 23, 38). Both *Mtb*MutT1 and *Msm*MutT1 hydrolyze 8-oxo-dGTP and 8-oxo-GTP to 8-oxo-dGDP and 8-oxo-GDP, respectively, preventing their misincorporation into nucleic acids (18, 19). Unlike *E. coli* MutT, or mycobacterial MutT2, MutT3, and MutT4, the mycobacterial MutT1 possesses an extra domain (CTD) with RHG motif, histidine phosphatase (18). Recent work from our lab revealed an unexpected role of NDK in escalating A to C mutations by converting 8-oxo-dGDP into 8-oxo-dGTP (8), raising a question if its (NDK) activity could be regulated.

In this study, comprehensive biochemical analyses reveal a protein phosphatase activity of mycobacterial MutT1 against NDK-*Pi* (Fig. 1A and B). This finding has been unprecedented in at least two ways. Firstly, so far, MutT proteins have been shown to possess phosphohydrolase activities against only small molecules. Mycobacterial MutT1 has also been shown to have activity on small molecules such as 8-oxo-dGTP and diadenosine polyphosphates (18, 19, 39). This is the first study that shows any MutT to work on a protein molecule. Secondly, so far, NDK has not been shown to be regulated by dephosphorylation of its His-*Pi* residue by any proteins. However, what has been even more surprising is that based on the presence of the RHG motif in MutT1 CTD, we anticipated CTD to possess this phosphatase activity. However, a mutation of the RHG motif to RAG did not suppress the phosphatase activity of mycobacterial MutT1 proteins against NDK-*Pi*. Instead, a mutation in the Nudix box of a critical Glu residue to Ala (E to A mutation) resulted in a complete loss of the MutT1 phosphatase activity against NDK-*Pi*. It was reported that the E53A mutation in the Nudix hydrolase motif in *E. coli* MutT results in loss of its phosphatase activity (26). Likewise, the E162A mutation in *M. smegmatis* MutT4 Nudix hydrolase motif failed to complement the MutT4 knockout strain (23). In *Msm*MutT1 and *Mtb*MutT1, these residues are represented by E81, and E69, respectively. Indeed, when these residues were mutated (E81A and E69A), the two MutT1 proteins lost their phosphatase activities even on *p*NPP (Fig. S2C and S3C). The His residue of the RHG motif (H170 for *Msm*MutT1 and H161 for *Mtb*MutT1) was mutated to Ala, not unexpectedly, with no loss of their phosphatase activities on *p*NPP (Fig. S2C and S3C). When the activity of MutT1 mutants was tested against NDK, we recorded a loss of *Msm*MutT1 E81A, and *Mtb*MutT1 E69A phosphatase activities against NDK-*Pi* (Fig. 2A and B), revealing the importance of Nudix hydrolase motif in dephosphorylation of even the proteins.

To confirm that only the MutT1 NTD (Nudix hydrolase motif) is responsible for its activity on NDK-*Pi*, we attempted to purify the *Msm*MutT1-NTD alone. Unfortunately, the NTD could not be purified with any reasonable purity. And, when we carried out a general phosphatase activity assay using *p*NPP as substrate, we did not detect any phosphatase activity in the preparation. This could be due to the instability of the NTD sequences when expressed independently of the CTD. We then turned to *Eco*MutT and *Msm*MutT2, which possess only the Nudix hydrolase motifs domain, and they both act on 8-oxo-dGTP. Moreover, the structures of these proteins are very similar to the MutT1 NTD structure (Fig. S9). When the phosphatase activities of *Eco*MutT and *Msm*MutT2 were checked against *p*NPP, high levels of phosphatase activities were detected (Fig. S5). However, when the activities of these were tested on *Eco*NDK-*Pi* and *Msm*NDK-*Pi*, respectively, neither of these dephosphorylated NDK-*Pi* (Fig. S6A and B). These observations then led us to ask the question of the role of the CTD of the mycobacterial MutT1 proteins. It is clear that the CTD (RHG motif) *per se* has no phosphatase activity on NDK-*Pi* (Fig. 2A and B). But the CTD may be required in some accessory ways for the NTD to act on NDK-*Pi*.

Our protein-protein interaction predictions (Fig. 3) suggested interactions between *Msm*NDK and *Msm*MutT1 CTD. To further support our hypothesis and validate the predictions, we generated a chimeric protein by replacing *Msm*MutT1 NTD with *Eco*MutT (*Eco-Msm*MutT1) (Fig. 4). We chose *Eco*MutT over *Msm*MutT2 to generate the chimeric protein because *Eco*MutT is efficient in hydrolyzing 8-oxo-dGTP into 8-oxo-dGMP (17, 40), whereas *Msm*MutT2 functions mainly as a dCTPase (20). The chimera showed an efficient phosphatase activity on *p*NPP when compared with *Msm*MutT1 (Fig. 4). Importantly, the chimeric protein led to efficient dephosphorylation of *Eco*NDK-*Pi* or *Msm*NDK-*Pi* (Fig. 5A and B). These observations further support that while MutT1 NTD plays the catalytic role of dephosphorylating NDK-*Pi*, the MutT1 CTD facilitates this activity.

**Fig 8:**
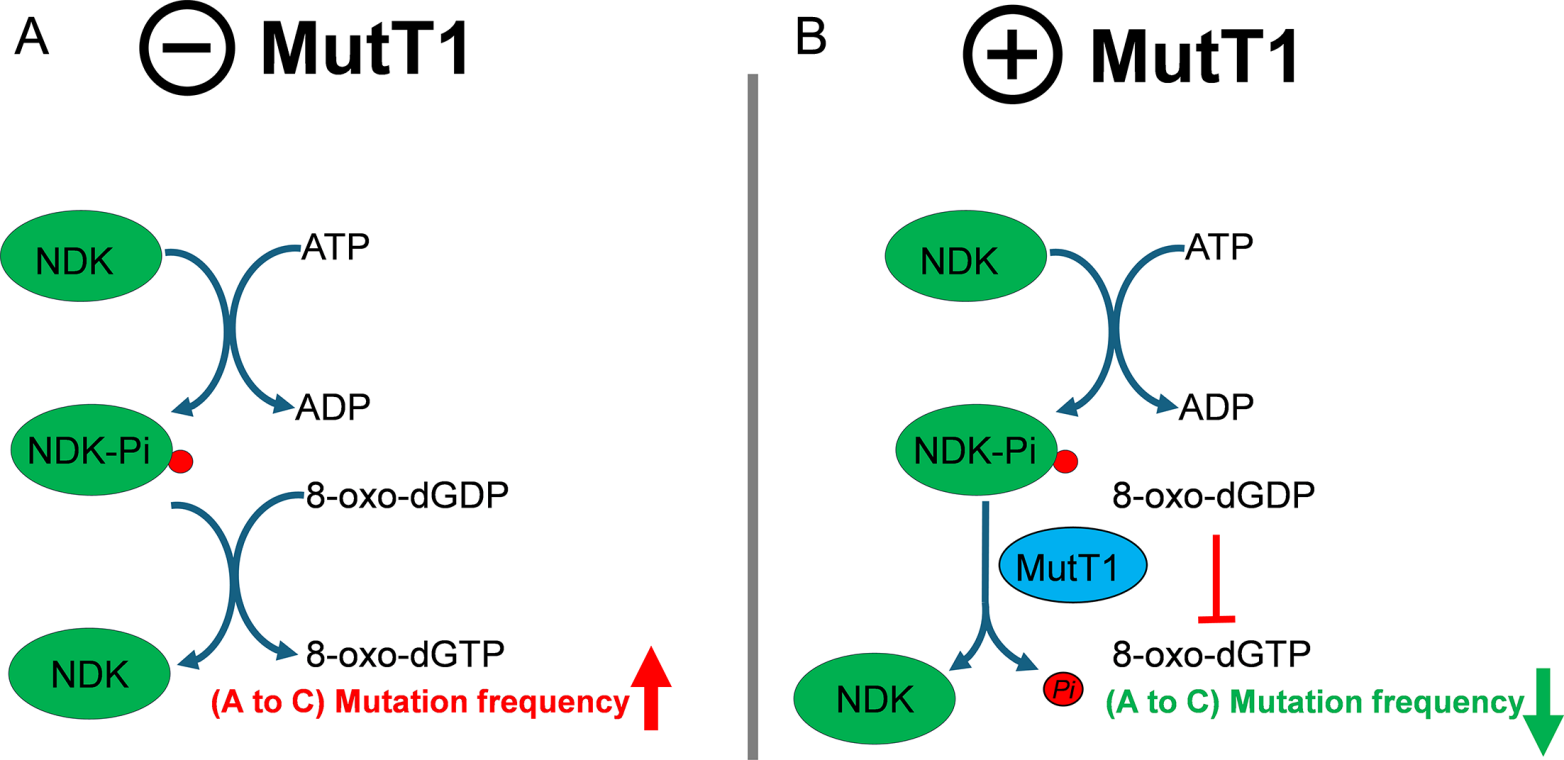
Model for the regulation of NDK activity by MutT1. (**A**) In the absence of MutT1, the active NDK-*Pi* catalyzes the conversion of 8-oxo-dGDP to 8-oxo-dGTP elevating A to C mutation frequency. (**B**) MutT1 dephosphorylates NDK-*Pi* preventing it from converting 8-oxo-dGDP to 8-oxo-dGTP resulting in a reduction of A to C mutation frequency.

To understand the effect of MutT1 in regulating the downstream roles of NDK (Fig. 6B through D), we made use of the fact that NDK catalyzes reversible phosphorylation of dNDPs to dNTPs (8, 41, 42). Our studies show that MutT1 efficiently prevents NDK-mediated conversion of ADP to ATP or 8-oxo-dGDP to 8-oxo-dGTP (Fig. 6B through D). No effect on ADP to ATP or 8-oxo-dGDP to 8-oxo-dGTP conversion was observed when NDK-*Pi* was treated with *Msm*MutT1 E81A or *Mtb*MutT1 E69A. The chimeric protein also behaved like the *Msm*MutT1 in preventing the conversion of the ADP to ATP. Furthermore, MutT1 regulates the downstream activity of NDK not only *in vitro* but also *in vivo* (Fig. 6 and 7). Particularly, the role of the mycobacterial MutT1 proteins in downregulating the mutation frequencies in *E. coli*, suggests an important role of its evolutionarily conserved presence in mycobacteria. Based on our investigations we propose a model (Fig. 8). This model shows how MutT1 regulates the activity of NDK. In the absence of MutT1, NDK-*Pi* catalyzes the reversible conversion of 8-oxo-dGDP to 8-oxo-dGTP elevating the (A to C) mutation frequency (Fig. 8A). On the other hand, the presence of MutT1 dephosphorylates NDK-*Pi* and prevents it from converting 8-oxo-dGDP to 8-oxo-dGTP resulting in reduction of (A to C) mutation frequency (Fig. 8B).

As we only tested the effect of MutT1 in maintaining the genome stability by regulating the activity of NDK, we cannot rule out the possibility that MutT1 might also regulate the other reported roles of NDK such as regulation of host defense mechanisms, interaction of NDK with FtsZ, bacterial growth, and signal transduction pathways (12, 43, 44).

Finally, the GO repair system comprising MutT, MutM (Fpg), and MutY has been suggested to be a multilayered system to protect the mycobacterial genomes from the oxidative damages inflicted by the ROS generated by the endogenous or the antibiotic-induced mechanisms (23, 25, 26). Moreover, with at least 4 MutT nudix hydrolases (possessing 8-oxo-dGTpase, dCTPase, and dATPase activities), 2 MutM (45, 46), and a distinct MutY (which removes not only A but also G and T paired against G, as well as A occurring against 8-oxo-G in DNA (29), mycobacteria possess an elaborate GO repair pathway. Importantly, now with the connection of MutT1 NTD in regulating the activity of NDK, which in turn regulates the homeostasis of the cellular nucleotide pool, it is evident that mycobacteria have evolved with an intricate mechanism of GO repair regulation.

## MATERIALS AND METHODS

### Bacterial strains, plasmids, enzymes, nucleotides, and DNA oligomers

Strains and plasmids used in this study are described in Table 1. DNA oligomers are listed in Table S1. Restriction enzymes (RE) and other DNA modifying enzymes used for cloning were ordered from New England Biolabs and Thermo Fisher Scientific. The 8-oxo-dGDP was purchased from Jena Bioscience, Germany. Media components were bought from BD Difco (Franklin Lakes, NJ).

**TABLE 1:** Description of strains and plasmids used in the study^*a*^

Strain/plasmid	Genotype	Reference/source
*E. coli* TG1	*supE* Δ(*lac-proAB*) *hsd*Δ*5F’* [*traD36 proAB+lacIq lacZΔM15*]	Novagen
*E. coli* BL21 Rosetta (DE3)	F' *ompT hsdSB(rB-mB-) gal dcm* (DE3) pRARE (CamR)	Novagen
JW0097 Δ*mutT*::*kan*	Δ *mutT790::kan*, LAM2 rph-1	(47)
*E. coli* M15/pREP4	*E. coli* strain with deletion of M15 in the lacZ gene. pREP4 codes for a repressor for leaky expression prevention	(48)
*E. coli* CC101	F’, *ara-600*, Δ*(gpt-lac)5*, *λ-*, *relA1*, *spoT1*, *thiE1*, F128- (used to screen for A to C or T to G mutation)	(49)
*E. coli* CC101Δ*ndk*::*kan* Δ*mutT*	CC101 Δ*mutT* cured strain, where *ndk* has been disrupted by introducing *kan* cassette	(8)
*M. smegmatis mc^2^155* MSMEG_2390(*mutT1*)::*hyg*	mc^2^155 strain where *mutT1* gene has been disrupted with *hyg* cassette	I. Kapoor and E. A. F. Emam, unpublished data
pBADHisB (pBAD)	*E. coli* expression vector harboring ColE1 ori of replication, *amp*^R^ marker, and arabinose inducible promoter	Invitrogen
pBAD *Eco* mutT	pBAD plasmid contains the Eco_mutT gene cloned (NcoI, NheI) under arabinose promoter	(20)
pTrcNdeHis-MsMutT1	pTrcNdeHis plasmid with *Msm mutT1 gene* cloned in its NdeI/HindIII site	(18)
pBAD *Msm* MutT1	Msm mutT1 was digested from pTrcNdeHis-MsMutT1 with (NcoI & HindIII) and subcloned into pBADHisB	This study
pACDH	A low copy number plasmid with ACYC ori of replication, compatible with the ColE1 origin of replication (TetR)	(50)
pACDH *Eco* ndk	pACDH plasmid contains the Eco_ndK gene cloned (NcoI, HindIII) under IPTG inducible promoter	This study
pJAM_His	Shuttle vector harboring ColE1 (*E. coli*) and pAL5000 (mycobacteria) origin of replications, *kan*^R^ marker, and mycobacterial acetamidase promoter	(51)
pJAM_*Msm* mutT1	Msm mutT1 was cloned into pJAM_His between BamHI and XbaI sites	This study
pJAM_*Msm* mutT1 E81A	pJAM with Msm mutT1 E81A mutant	This study
pJAM_*Msm* mutT1 H170A	pJAM with Msm mutT1 H170A mutant	This study
pJAM_*Mtb* mutT1	Mtb mutT1 was cloned into pJAM_His in XbaI site	This study
pJAM_*Mtb* mutT1 E69A	pJAM with Mtb mutT1 E69A mutant	This study
pJAM_*Mtb* mutT1 H161A	pJAM with Mtb mutT1 H161A mutant	This study
pTrc*Msm-mutT2*	pTrc99c plasmid with *Msm-mutT2* cloned in its NcoI and HindIII sites (from pET14b*Msm-mutT2*)	(20)
pPROEX-HTb *Eco-Msm*MutT1 chimera	Chimera (Eco mutT + Msm mutT1 CTD) was cloned into pProEx-Htb between BamHI and XhoI sites	This study
pBAD_*Eco_ndk*	A pBAD plasmid having *Eco_ndk* cloned (NcoI, BglII) under arabinose promoter	(8)
pPROEX-HTb *Msm* ndk	Msm ndk gene was cloned into pProEx-Htb between NcoI and HindIII sites	This study
pQE30*Mtb-ndk*	Mtb *ndk* gene was cloned into pQE30 between *Bam*HI and *Hind*III	(30)
pBAD *Msm* mutT1 E81A	pBAD plasmid with Msm mutT1 E81A mutant	This study
pBAD*Eco-Msm*MutT1 chimera	pPROEX chimera was digested with NcoI & XhoI and the chimera was subcloned into pBADHisB	This study

^*a*^ Isopropyl β-D-thiogalactopyranoside, IPTG.

### Media and growth conditions

*E. coli* strains were grown in Luria-Bertani (LB) medium. For growth on a solid surface, 1.6% agar was added to the LB medium. Minimal medium plates used for (A to C) reversion assays contained 1× M9 salts (47 mM Na_2_HPO_4_, 22 mM KH_2_PO_4_, 8.5 mM NaCl, and 18.6 mM NH_4_Cl), 2 mM MgSO_4_, 0.1 mM CaCl_2_, 5 µg/mL thiamine, 0.2% glucose (or lactose), and 1.6% agar. Media were supplemented with ampicillin (Amp), hygromycin (Hyg), kanamycin (Kan), and tetracycline (Tet) as required at concentrations of 100 µg/mL, 150 µg/mL, 25 µg/mL, and 7.5 µg/mL, respectively, unless mentioned otherwise. For culturing *M. smegmatis* strains, LB media containing 0.2% tween 80 (vol/vol) (LBT) was used. For growing *M. smegmatis* strains on solid media, LB containing 0.04% tween 80 (vol/vol) was supplemented with 1.5% agar (wt/vol). Media were supplemented with Kan (50 µg/mL) and Hyg (50 µg/mL), as required.

### Cloning of *Msm*MutT1

*Msm*MutT1 (MSMEG_2390) open reading frame (ORF) was amplified from the *M. smegmatis* mc^2^155 genomic DNA using *Msm* mutT1 BamH1 forward primer (Fp) and *Msm* mutT1 Xba1 reverse primer (Rp) primers. The PCR was incubated at 94°C for 5 min followed by 30 cycles of incubations at 94°C for 1 min, 63°C for 30 s, 72°C for 2 min, and then 72°C for 5 min. The PCR product was purified, digested with BamHI and XbaI, and cloned into similarly digested pJAM_His. The clones were confirmed by RE digestions and DNA sequencing.

### Cloning of *Mtb*MutT1

*Mtb*MutT1 (Rv2985) ORF was amplified from *M. tuberculosis* H37Rv genomic DNA using *Mtb* mutT1 Xba1 Fp and *Mtb* mutT1 Xba1 Rp. The PCR was incubated at 94°C for 5 min followed by 30 cycles of heatings at 94°C for 1 min, 60°C for 30 s, and 72°C for 2 min, and then at 72°C for 5 min. The PCR product was purified, digested with XbaI, cloned into similarly digested pJAM_His, and confirmed by RE digestions and DNA sequencing.

### Cloning of *Msm*NDK

The ORF of *Msm*NDK (MSMEG_4627) was amplified from *M. smegmatis* mc^2^155 genomic DNA using *Msm* ndK-Ncoi-Fp and *Msm* ndK-Hindiii-Rp. The PCR was incubated at 94°C for 5 min followed by 30 cycles of incubations at 94°C for 1 min, 58°C for 30 s, and 72°C for 1 min, and then at 72°C for 5 min. The purified PCR product was digested with NcoI and HindIII and cloned into similarly digested pProEx-Htb. The clone was confirmed by RE digestions and DNA sequencing.

### Generation of MutT1 mutants

*Msm*MutT1 (E81A or H170A), and *Mtb*MutT1 (E69A or H161A) were generated by site-directed mutagenesis (SDM), using pJAM*Msm*MutT1 and pJAM*Mtb*MutT1 templates, respectively. The primer combinations of *Msm* mutT1 E81A Fp and *Msm* mutT1 E81A Rp; *Msm* mutT1 H170A Fp and *Msm* mutT1 H170A Rp; *Mtb* mutT1 E69A Fp and *Mtb* mutT1 E69A Rp; *Mtb* mutT1 H161A Fp, and *Mtb* mutT1 H161A Rp were used. The reactions with 100 ng template DNA, 10 pmols each of the forward and reverse primers, 250 µM dNTPs, 1× Q5 polymerase buffer, and Q5 DNA polymerase enzyme (NEB #M0491) were heated at 98°C for 1 min followed by 20 cycles of incubations at 98°C for 20 s, 68°C for 30 s, and 72°C for 6 min and final extension at 72°C for 10 min. The PCR product was digested with DpnI (Thermo Scientific # ER1702) and used to transform *E. coli* TG1. Plasmids from the transformants were confirmed for SDM by DNA sequencing.

### Chimeric *Eco-Msm*MutT1 generation

The chimera generation was done in four steps. Firstly, *Eco*MutT was amplified from pBAD *Eco*MutT using the *Eco* mutT BamHI Fp and *Eco* mutT chimera Rp. The PCR was incubated at 94°C for 3 min followed by 30 cycles of incubations at 94°C 1 min, 65°C 30 s, 70°C 1 min 30 s followed by a final extension at 70°C for 5 min. Secondly, *Msm*MutT1 CTD was amplified from pJAM *Msm*MutT1 using *Msm* mutT1 CTD Fp and *Msm* mutT1 CTD XhoI Rp. The PCR was incubated at 94°C for 3 min followed by 30 cycles of incubations at 94°C for 1 min, 65°C for 30 s, 70°C for 1 min 30 s followed by a final extension at 70°C for 5 min. Thirdly, for the overlapping PCR, PCR products *Eco*MutT and *MsmMutT1*-CTD were used to generate a full-length fragment. The PCR reaction was incubated at 94°C for 3 min followed by 12 cycles of incubations at 94°C 1 min, 56°C 20 min, 70°C 1 min followed by a final extension at 70°C 10 min. Fourthly, for the chimera cloning, the PCR product from the third step was used as a template to amplify the chimeric gene using *Eco* mutT BamHI Fp and *Msm* mutT1 CTD XhoI Rp. The PCR was incubated at 98°C for 30 s followed by 30 cycles of incubations at 98°C for 10 s, 65°C for 30 s, 72°C for 35 s, and a final extension at 72°C for 2 min. The PCR product was purified, digested with BamHI and XhoI, and cloned into similarly digested pProEx-Htb. The clones were confirmed by RE digestions and DNA sequencing.

### Purification of MutT1 proteins

MutT1 proteins were purified from *M. smegmatis* mc^2^155 Δ*mutT1* strain. Briefly, the expression plasmids containing MutT1 constructs were electroporated into mc^2^155 Δ*mutT1*. An isolated colony was inoculated in LB with 0.2% tween 80 and Kan. Inoculum (1% of the saturated culture) was sub-cultured into 3 L LB with 0.2% tween 80 and Kan. At OD_600_ of 0.6, protein expression was induced with 1% acetamide, and the culture was further incubated at 30°C for 9 h. The cells were pelleted by centrifugation, resuspended in buffer A (20 mM Tris-HCl, pH 8.0, 1 M NaCl, 10% [vol/vol] glycerol, 20 mM imidazole, and 2 mM β-mercaptoethanol), lysed by sonication and ultracentrifuged at 26 K rpm at 4°C for 2 h (using optima XPNs Ultra Centrifuge, Beckman Coulter). The clarified lysate was loaded onto a pre-equilibrated Ni-NTA column, and washed with wash buffer (1 M NaCl, 20 mM Tris-HCl pH 8, 10% [vol/vol] glycerol, 2 mM β-mercaptoethanol, and 40 mM imidazole). The proteins were eluted with a gradient of 40 to 1,000 mM imidazole in a wash buffer lacking imidazole. The fractions containing the desired protein were pooled, concentrated, and loaded onto a Superdex-75 column, and eluted with gel filtration buffer (1 M NaCl, 20 mM Tris-HCl pH 8, 10% [vol/vol] glycerol, and 2 mM β-mercaptoethanol). The fractions containing the purified proteins were pooled and dialyzed against dialysis buffer (750 mM NaCl, 20 mM Tris-HCl pH 8, 10% [vol/vol] glycerol, and 2 mM β-mercaptoethanol), followed by dialysis in storage buffer (500 mM NaCl, 20 mM Tris-HCl pH 8, 50% glycerol, and 2 mM β-mercaptoethanol) and stored at −20°C.

### Purification of *Msm*NDK

*Msm*NDK was expressed and purified using pPROEX-HTb *Msm*NDK. Briefly, *E. coli* M15 was transformed with pPROEX-HTb *Msm*NDK. A single isolated colony was used to inoculate for starter culture in 25 mL LB containing Kan and Amp. Saturated culture (1%) was used to inoculate 2 L LB with Kan and Amp, and incubated under shaking at 37°C. When the culture reached an OD_600_ of ~0.6, cells were induced with 1 mM IPTG and incubated further at 37°C for 4 h. Cells were pelleted at 8 K RPM at 4°C for 5 min using Kubota 6500 centrifuge and resuspended in buffer A [500 mM NaCl, 50 mM Tris-HCl pH 8, 10% vol/vol glycerol, 10 mM imidazole, 2 mM β-mercaptoethanol, and 1 mM phenylmethylsulfonyl fluoride (PMSF)]. Resuspended cells were sonicated (2 s on/2 s off cycles for 1 min at 35% amplitude), and centrifuged at 13 K at 4°C for 30 min. The supernatant was then subjected to ultracentrifugation at 26 K RPM at 4°C for 2 h (using optima XPNs Ultra Centrifuge, Beckman Coulter). Cell-free extract so prepared was loaded onto 1 mL pre-equilibrated Ni-NTA column. The column was washed with 50 mM imidazole. Elution of proteins was done using a gradient of 10 mM to 1 M imidazole in 30 mL buffer A (without imidazole). Fractions were loaded on 12% SDS-PAGE. The fractions containing the desired protein were pooled, concentrated, and loaded onto a Superdex-75 column, and eluted with gel filtration buffer (500 mM NaCl, 20 mM Tris-HCl pH 8, 10% [vol/vol] glycerol, and 2 mM β-mercaptoethanol). Fractions enriched with the protein were dialyzed against 50% glycerol, 150 mM NaCl, 50 mM Tris-HCl pH 8, and stored at −20°C.

### Purification of *Eco-Msm*MutT1 chimera

*E. coli* BL21 was transformed with pPROEX-HTb-*Eco-Msm*MutT1, and an isolated colony was inoculated in 25 mL LB containing Amp. Saturated culture (1%) was used to inoculate 2 L LB, and incubated under shaking at 37°C for 3 h. Cells were induced with 1 mM IPTG and grown further at 25°C for 8 h. Cells were pelleted at 8 K RPM, at 4°C for 5 min, resuspended in buffer A (1,000 mM NaCl, 50 mM Tris-HCl pH 8, 10% [vol/vol] glycerol, 10 mM imidazole, 2 mM β-mercaptoethanol, and 1 mM PMSF), sonicated (2 s on/2 s off cycles, for 1 min at 35% amplitude), and centrifugated at 13 K, 4°C for 30 min. The supernatant was subjected to ultracentrifugation at 26 K rpm at 4°C for 2 h (using optima XPNs Ultra Centrifuge, Beckman Coulter). Cell lysate was loaded into a 1 mL pre-equilibrated Ni-NTA column. After loading, the column was washed with 50 mM imidazole. Proteins were eluted using a gradient of 10 mM to 1 M imidazole in 30 mL buffer A without imidazole. Fractions were analyzed on 12% SDS-PAGE, and those containing the desired protein were pooled, concentrated, loaded onto Superdex-75 column, and eluted with 1 M NaCl, 20 mM Tris-HCl pH 8, 10% (vol/vol) glycerol and 2 mM β-mercaptoethanol. Fractions enriched for the desired protein were dialyzed against 50% glycerol, 500 mM NaCl, 50 mM Tris-HCl, pH 8, and stored at −20°C.

### Purification of *Mtb*NDK, *Eco*NDK, *Eco*MutT, and *Msm*MutT2

The *Mtb*NDK, *Eco*NDK, *Eco*MutT, and *Msm*MutT2 were purified by Ni–NTA affinity chromatography followed by size-exclusion chromatography using a Superdex 75 column. The culture growth and protein purifications were done essentially as described (8, 20, 30).

### General phosphatase activity assay

The assays were performed as described (52). Briefly, purified protein (1 µg) was incubated with 10 mM *para*-nitrophenylphosphate (*p*NPP) in a 50 µL reaction consisting of 50 mM Tris-HCl pH 7.5, 25 mM NaCl, and 100 mM MgCl_2_ at 37°C for 30 min. The reactions were stopped by adding 150 µL 1 N NaOH. The absorbance at 405 nm was read in a Tecan plate reader.

### Preparation of NDK-*Pi* and its use in dephosphorylation assays

Briefly, 1 µg of purified NDK was incubated in kinase buffer (50 mM Tris-HCl, pH 8.0, 50 mM KCl, 10 mM MgCl_2_) containing 1 µCi of γ-^32^P-ATP (3,000 Ci/mM) at 30°C for 1 h to generate NDK-*Pi*. Further incubations with 1 to 2 µg MutT1 proteins were done for 1 h at 30°C. The reactions were terminated by adding 1× SDS-PAGE sample buffer (without heating) and resolved on 12% SDS-PAGE. After electrophoresis, the gel was exposed to a phosphor imager screen (Fujifilm Bas cassette2, Japan) for 4 h followed by imaging with Typhoon 9210 phosphor imager (GE Healthcare, USA) and Azure Sapphire (Azure Biosystems, USA).

### ADP to ATP, or 8-oxo-dGDP to 8-oxo-dGTP conversion assay

Briefly, purified NDK (1 µg) was incubated in kinase buffer (50 mM Tris-HCl, pH 8.0, 50 mM KCl, 10 mM MgCl_2_) containing 1 µCi of γ-^32^P-ATP (3,000 Ci/mM) at 30°C for 1 h. Further incubation was with 1 to 2 µg MutT1 proteins for 1 h at 30°C. ADP, or 8-oxo-dGDP were added to the reaction to 1 mM and incubated at 30°C for 5 min. The reaction was stopped by 2% (final concentration) HCOOH. The reaction aliquots (2.5 µL) were spotted on PEI Cellulose F plate (MERCK # 105579), developed with 1.5 M KH_2_PO_4_ (pH 3.4), dried, exposed to phosphor imager screen (Fujifilm Bas cassette2, Japan) for 4 h, and imaged on Typhoon 9210 phosphor imager (GE Healthcare, USA) and Azure Sapphire (Azure Biosystems, USA).

### A to C reversion assay

*E. coli* CC101 and CC101Δ*mutT*Δ*ndk* were transformed with relevant plasmids, and grown on LB agar containing 50 µg/mL 5-bromo-4-chloro-3-indolyl β-D-galactopyranoside (X-Gal) and suitable antibiotics. Isolated white colonies were inoculated into 2 mL LB having 0.005% arabinose, 100 µM IPTG, desired antibiotics, and grown at 37°C for 16 h. OD_600_ was normalized to 1.5 for each replicate, and a 20 µL aliquot was spread on an M9 plate containing 0.2% lactose to determine Lac^+^ revertants. Aliquots (20 µL) of 10^−5^ dilution were spread on M9 agar containing 0.2% glucose to determine the total viable counts. The plates were incubated at 37°C for 24 h (glucose plates) and 48 h (lactose plates). The reversion frequencies were obtained by dividing the colony numbers observed on the lactose plate by those on the glucose plates for the number of replicates indicated in the figure legends.

## Data Availability

The data underlying this article will be shared on reasonable request to the corresponding author.

## SUPPLEMENTAL MATERIAL

Supplemental table and figures jb.00314-24-s0001.pdf Table S1; Fig. S1 to S9.

ASM does not own the copyrights to Supplemental Material that may be linked to, or accessed through, an article. The authors have granted ASM a non-exclusive, world-wide license to publish the Supplemental Material files. Please contact the corresponding author directly for reuse.
