# Supplementary material for: An unusual activity of mycobacterial MutT1 Nudix hydrolase domain as a protein phosphatase regulates nucleoside diphosphate kinase function
Source: J Bacteriol. 2024 Dec 11;207(1):e00314-24. doi: 10.1128/jb.00314-24 (PMC11784022; doi:10.1128/jb.00314-24)
Supplement: Supplemental table and figures — Table S1; Fig. S1 to S9. [file jb.00314-24-s0001.pdf]

Table S1. List of DNA oligomers used in the study.

DNA oligomer	Sequence (5' to 3')
Msm mutT1 BamH1 Fp	ACATGGGATCCGTGATGCCGGTGGAC
Msm mutT1 Xba1 Rp	AGGCTCTAGACTTCTCGTCGGGAGGG
Mtb mutT1 Xba1 Fp	ACCTTCTAGAGTGTCGATCCAGAAC
Mtb mutT1 Xba1 Rp	AGGCTCTAGAGGCCCGCACGTTGGC
Msm mutT1 E81A Fp	GCACGCGCGATCCACGAGGAGAC
Msm mutT1 E81A Rp	GTGGATCGCGCGTGCCGCGGCCAC
Mtb mutT1 E69A Fp	GTGCGGGCGATACTCGAGGAGAC
Mtb mutT1 E69A Rp	GAGTATCGCCCGCACCGCCCCCAC
Msm mutT1 H170A Fp	TGCGGGCCGGCACGGCCGGGCG
Msm mutT1 H170A Rp	TGCCGGCCCGCACGACGAGTACC
Mtb mutT1 H161A Fp	TGCGGGCTGGCACCGCGGGCAG
Mtb mutT1 H161A Rp	TGCCAGCCCGCACCACCAGCACC
Eco mutT BamHI Fp	GTCGGATCCATGAAAAAGCTGCAA
Eco mutT chimera Rp	CCGGTCGTTTCAGACGTTTA
Msm mutT1 CTD Fp	TAAACGTCTGAAACGACCGG
Msm mutT1 CTD Xhoi Rp	AGCGCTCGAGTTACTTCTCGTCG
Msm ndK-Ncoi-Fp	GCGCCATGGTGACTGAGCGGACCCTCGTA
Msm ndK-Hindiii-Rp	AGTAAGCTTTCAGGCGGTGGCCTCGCCGG

Figure S1

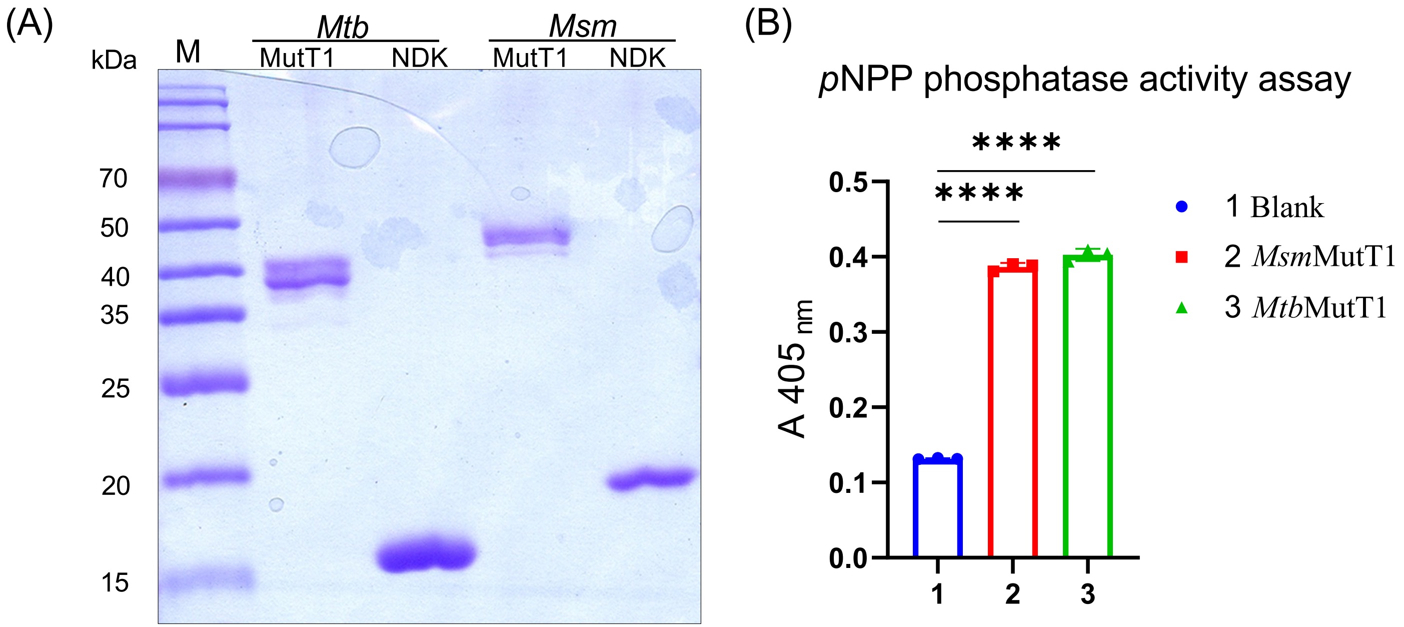

Figure. S1: Purification of NDK and MutT1 proteins, and the general phosphatase activities of MutT1 proteins using *p*NPP. (i) 15% SDS-PAGE gel analysis showing the quality of the purified proteins (*Mtb*MutT1, *Mtb*NDK, *Msm*MutT1 and *Msm*NDK). The calculated molecular masses of *Mtb*MutT1, *Mtb*NDK, *Msm*MutT1 and *Msm*NDK with His_6 tag are 37.5, 16.7, 39 and 18.1 kDa, respectively. (ii) The graph illustrates the general phosphatase activities of the MutT1 proteins using *p*NPP as a substrate. The substrate was incubated with either water (bar 1), 1 µg *Msm*MutT1 (bar 2), or 1 µg *Mtb*MutT1 (bar 3). Bars represent mean ± SD for n = 3. *p* values, $* p < 0.05$; $** p < 0.01$; $*** p < 0.001$ indicate significant differences between samples; 'ns' represent not significant. One-way ANOVA method was used to calculate *p* value.

Figure S2

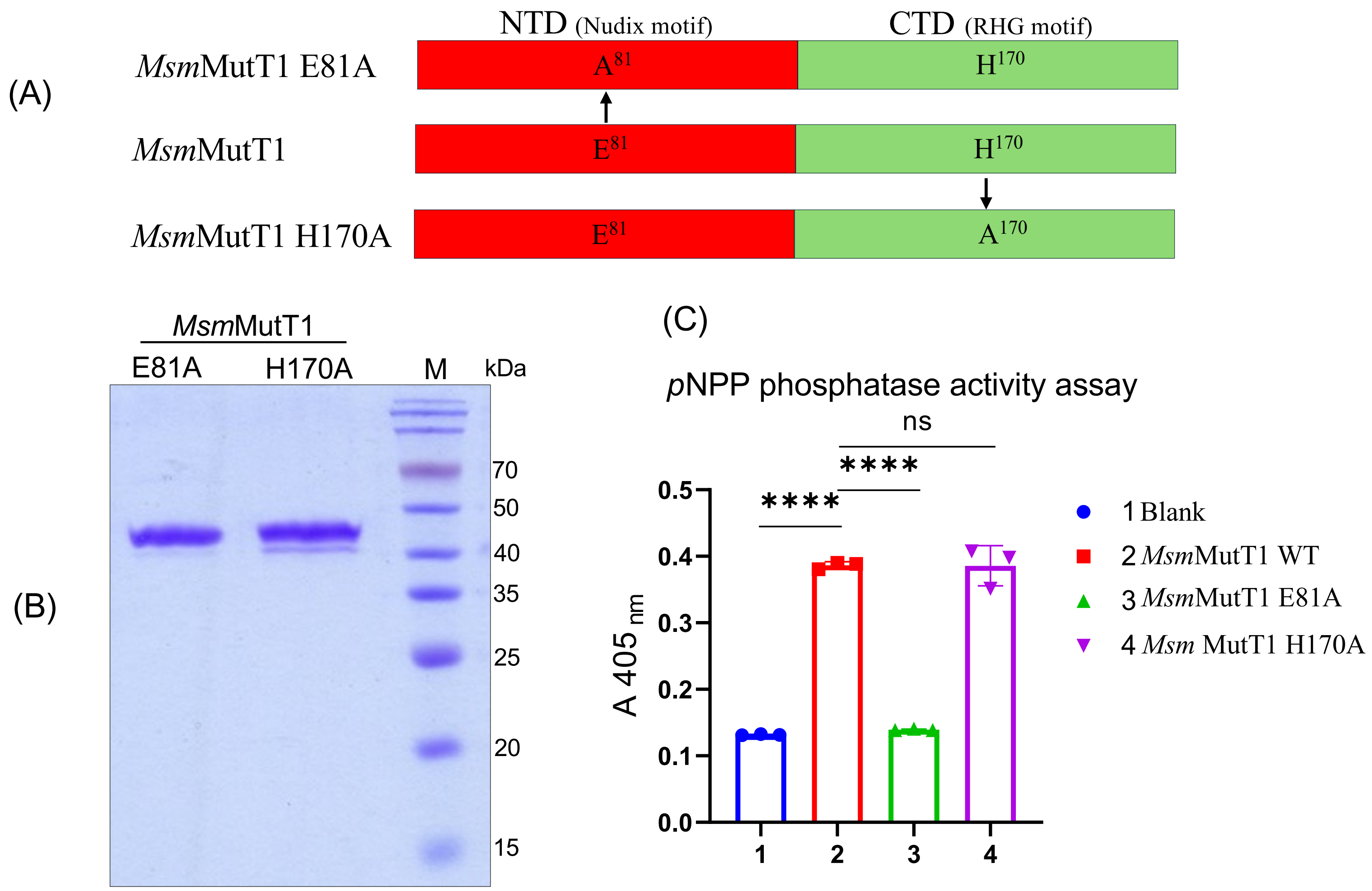

Figure S2: Purification of *Msm*MutT1 mutant proteins and their phosphatase activity. (A) A schematic depicting *Msm*MutT1 with its two domains, and the positions where the mutations were generated. (B) Analysis on 15% SDS-PAGE shows the quality of the purified proteins. ~3 µg of *Msm*MutT1 E81A and *Msm*MutT1 H170A were loaded into 15% SDS-PAGE gel. The calculated molecular mass of the proteins is ~39 kDa. (C) The graph illustrates the general phosphatase activity of MutT1 proteins using *p*NPP as a substrate. The substrate was incubated with either water (bar 1), 1 µg *Msm*MutT1 (bar 2), 1 µg *Msm*MutT1 E81A (bar 3) or 1 µg *Msm*MutT1 H170A (bar 4). Bars represent mean ± SD for n = 3. *p* values, ∗ $p < 0.05$; ∗∗ $p < 0.01$; ∗∗∗ $p < 0.001$ indicate significant differences between samples; 'ns' represent not significant. One-way ANOVA method was used to calculate *p* value.

Figure S3

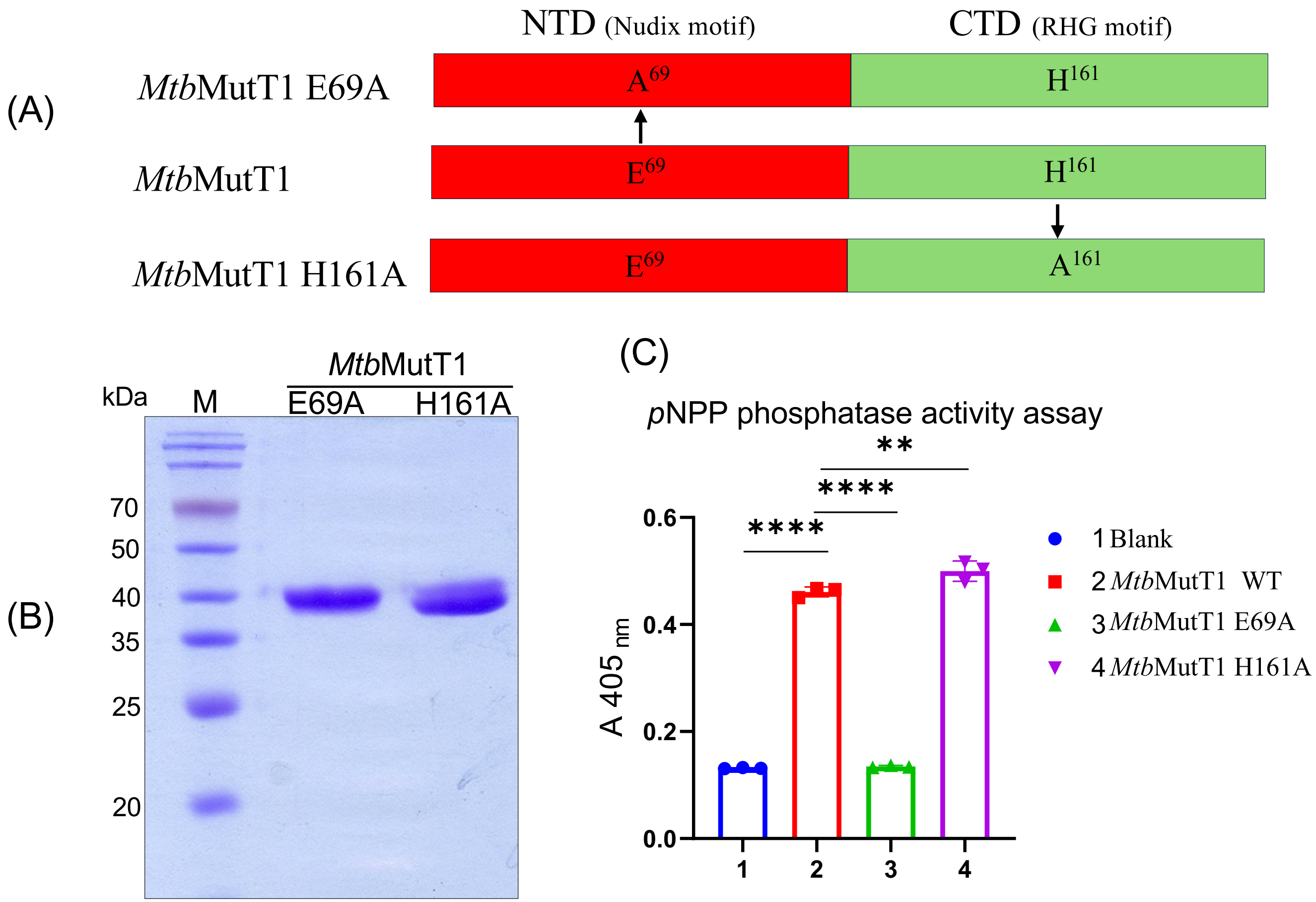

Figure S3: Purification of *Mtb*MutT1 mutant proteins and their phosphatase activity. (A) A schematic depicting *Mtb*MutT1 with its two domains, and the positions where the mutations were generated. (B) Analysis of the proteins on 15% SDS-PAGE gel showing the quality of the purified proteins. ~3 µg each of *Mtb*MutT1 E69A and *Mtb*MutT1 H161A were loaded into 15% SDS PAGE gel. The calculated molecular mass of the proteins is ~37 kDa. (C) The graph illustrates the general phosphatase activity of MutT1 proteins using *p*NPP as a substrate. The substrate was incubated with either water (bar 1), 1 µg *Mtb*MutT1 (bar 2), 1 µg *Mtb*MutT1 E69A (bar 3) or 1 µg *Mtb*MutT1 H161A (bar 4). Bars represent mean ± SD for n = 3. *p* values, $*$ $p < 0.05$; $**$ $p < 0.01$; $***$ $p < 0.001$ indicate significant differences between samples; 'ns' represent not significant. One-way ANOVA method was used to calculate *p* value.

Figure S4

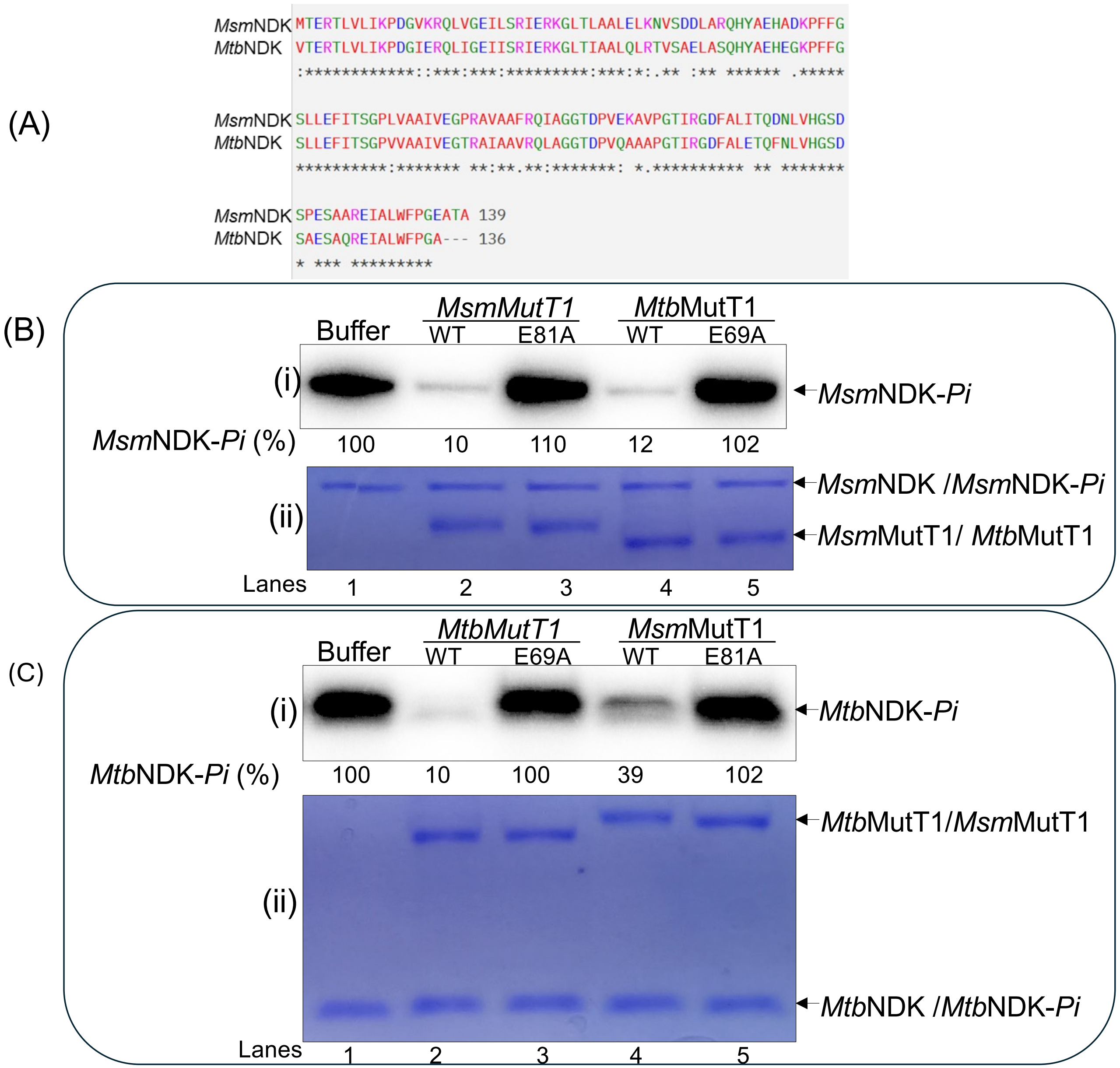

Figure S4:Dephosphorylation of NDK-Pi with mycobacterial MutT1 (A) Sequence alignment of NDK proteins from *M. smegmatis* (*Msm*NDK) and *M. tuberculosis (Mtb*NDK*).* Sequences were obtained from Mycobrowser website [46] and analysed using Clustal Omega Multiple Sequence Alignment (MSA) [47]. (B) Dephosphorylation of *Msm*NDK-Pi by *Msm*MutT1 (WT or E81A) and *Mtb*MutT1 (WT or E69A). *Msm*NDK-Pi (1 µg) was incubated with either buffer alone; 1 µg of *Msm*MutT1, 1 µg *Msm*MutT1 E81A, 1 µg *Mtb*MutT1 WT, or 1 µg *Mtb*MutT1 E69A (lanes 1-5, respectively). The reactions were incubated at 30 ° C for 1 h, mixed with 5 µL SDS-PAGE sample buffer and loaded (without heating) onto 12% SDS-PAGE. Gels were subjected to phosphor imaging, fixed, and stained with Coomassie brilliant blue (CBB). Panels (i) and (ii) represent autoradiogram, and CBB stained gel, respectively. Values of *Msm*NDK-Pi (%) with reference to lane 1 are shown below panel i. (C) Dephosphorylation of *Mtb*NDK-Pi by *Mtb*MutT1 (WT or E69A) and *Msm*MutT1 (WT or E81A). *Mtb*NDK-Pi (1 µg) was incubated with either buffer alone (lane 1); 1µg of *Mtb*MutT1, 1 µg *Mtb*MutT1 E69A, 1 µg *Msm*MutT1, or 1 µg *Msm*MutT1 E81A (lanes 1-5, respectively). The reactions were processed as in (B). Panels: (i) autoradiograms (ii) CBB stained gel. Values of *Msm*NDK-Pi (%) with reference to lane 1 are shown below panel i. Because of no heating of the samples after adding sample dye, *Msm*NDK (hexamer) migrates much slower than its expected monomeric molecular weight (also refer to Fig. S7). 2- *Msm*MutT1E81A migrates slightly faster than *Msm*MutT1 WT, while *Mtb*MutT1E69A migrates slower than *Mtb*MutT1 WT. This was only observed when samples are not heated.

Figure S5

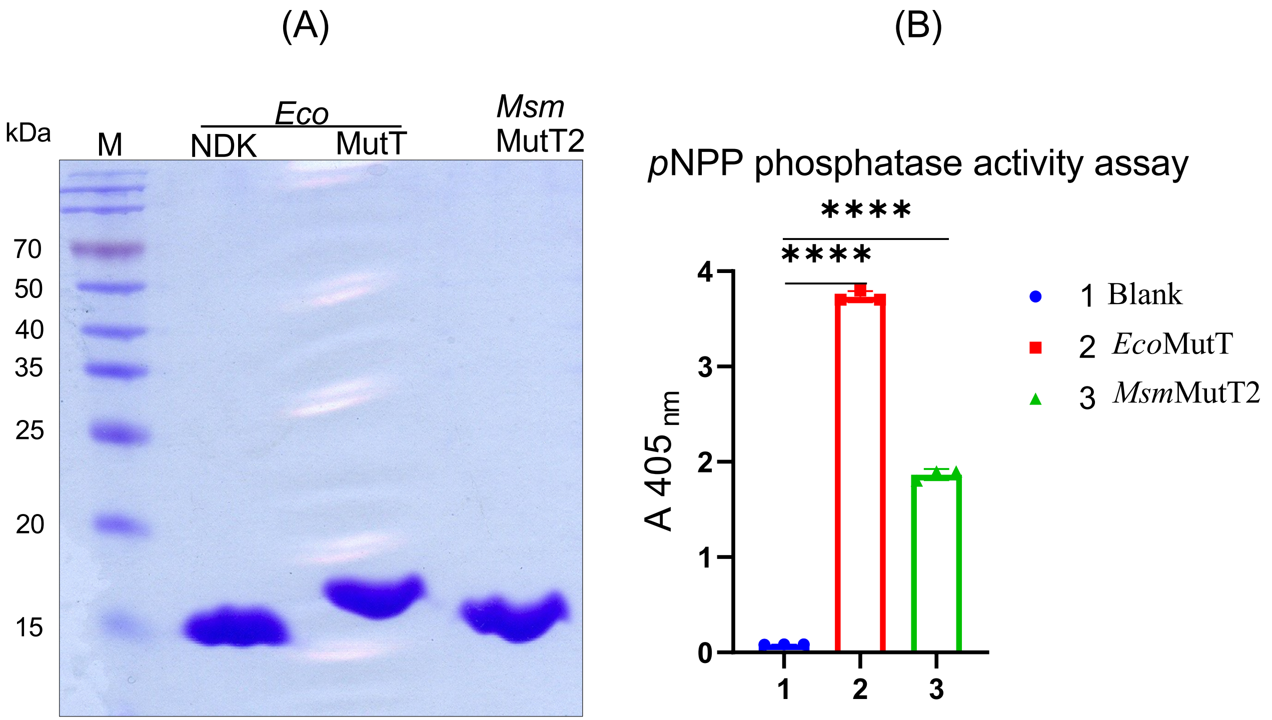

Figure S5: Purification of *Eco*NDK, *Eco*MutT and *Msm*MutT2 proteins and the phosphatase activity of MutT proteins. (A) Analysis on 15% SDS-PAGE gel showing quality of the purified proteins. ~5 µg of *Eco*NDK, *Eco*MutT and *Msm*MutT2, were loaded into 15% SDS PAGE gel. The calculated molecular masses of *Eco*NDK, *Eco*MutT and *Msm*MutT2 with His_6 tag are ~16.5, ~17 and ~14.8 kDa, respectively. (B) The graph illustrates the general phosphatase activity of MutT proteins using *p*NPP as substrate. The substrate was incubated with either water (bar 1), 1 µg *Eco*MutT (bar 2) or 1 µg *Msm*MutT2 (bar 3). Bars represent mean ± SD for n = 3. *p* values, * $p < 0.05$; ** $p < 0.01$; *** $p < 0.001$ indicate significant differences between samples; 'ns' represent not significant. One-way ANOVA method was used to calculate *p* value.

Figure S6

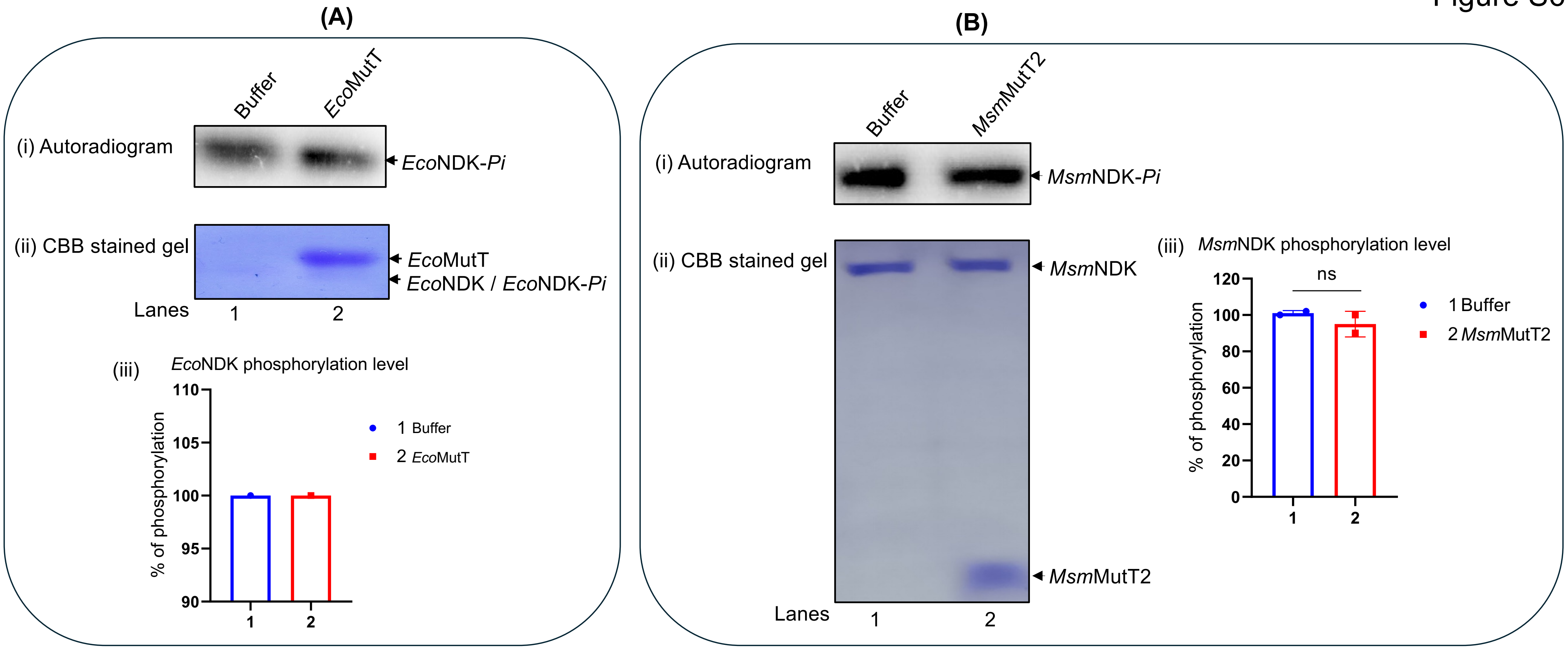

Figure S6: Treatments of NDK-*Pi* with *Eco*MutT and *Msm*MutT2. (A) Treatment of *Eco*NDK-*Pi* with *Eco*MutT. The *Eco*NDK-*Pi* (1 µg) was incubated with buffer alone (lane 1) or *Eco*MutT (2 µg) (lane 2). Panels (i) and (ii) represent autoradiogram and CBB stained gel. (iii) represents quantification of *Eco*NDK-*Pi* levels. **(B) Treatment of *Msm*NDK-*Pi* by *Msm*MutT2.** The *Msm*NDK-*Pi* (1 µg) was incubated with buffer alone (lane 1) or 1 µg *Msm*MutT2 (lane 2). Panels (i) and (ii) represent autoradiogram and CBB stained gel. (iii) represents quantification of *Msm*NDK-*Pi* levels. It may be noted that because the samples are not heated in the sample buffer prior to loading in the SDS-PAGE, *Msm*NDK migrates slower than its expected monomeric molecular weight (also refer to Fig. S7).

Figure S7

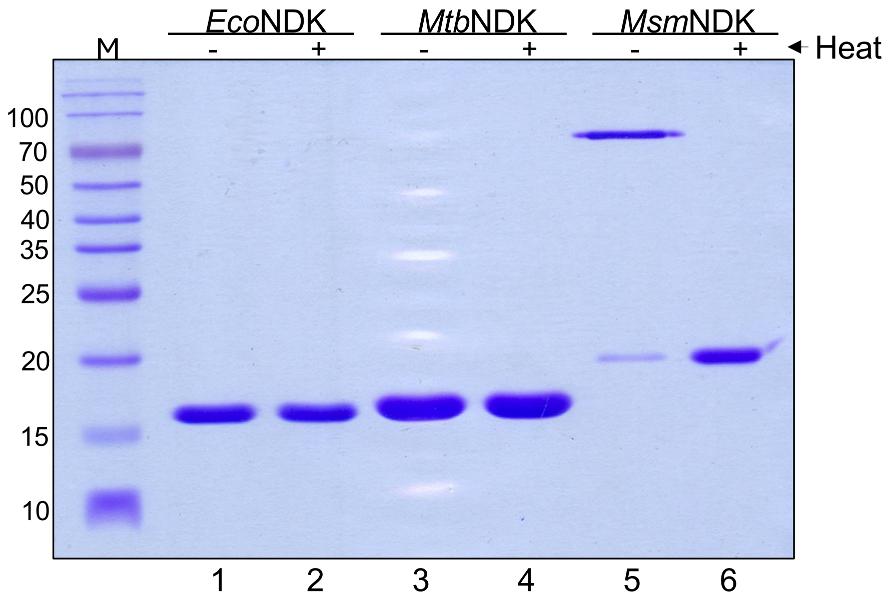

Figure S7: Effect of heating in SDS-sample buffer on migration of NDK proteins. Analysis on 12% SDS-PAGE gel showing the effect of heating on the migration of NDK proteins. 1X SDS dye was added to ~2 µg of NDK proteins. Samples were untreated or treated with heat at 90 ºC for 10 min and then loaded onto 12% SDS-PAGE gel. The calculated molecular masses of *Eco*NDK, *Mtb*NDK and *Msm*NDK with His tag are ~16.5, ~16.7 and ~18.1 kDa respectively. Only *Msm*NDK was shown to migrate slowly in unheated condition.

Figure S8

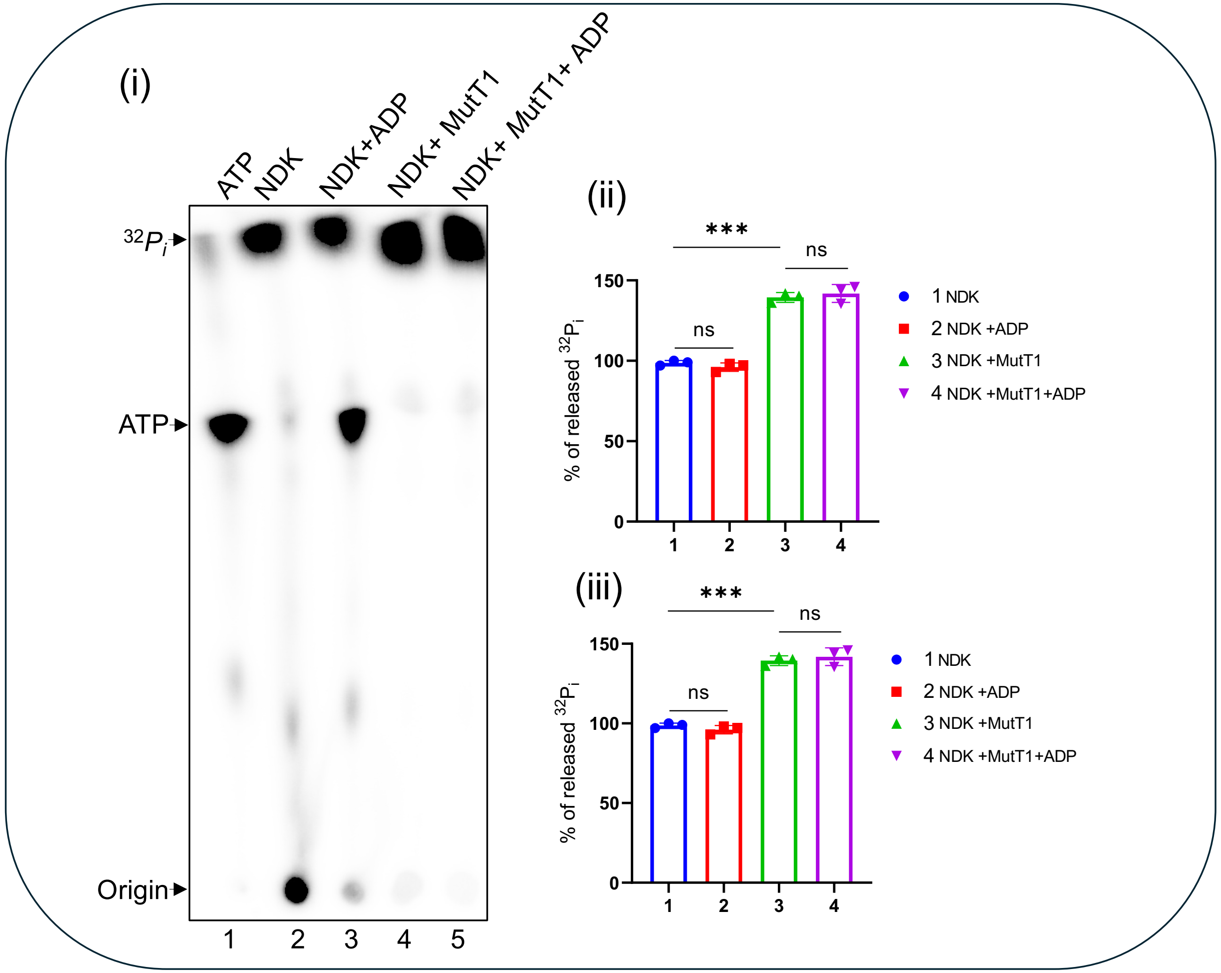

Figure S8: (A) ADP to ATP conversion by *Msm*NDK. *Msm*NDK-Pi (1 µg) was incubated with buffer (lane 2 &3), 2 µg *Msm*MutT1 (lane 4 &5), at 30 oC for 1 h followed by addition of ADP to sample (3 and 5) for 5 minutes as described in Materials and Methods. For control radiolabelled ATP was spotted in lane 1. (ii) represents % of ADP to ATP conversion by NDK. (iii) represents % of released ^{32}Pi.

Figure S9

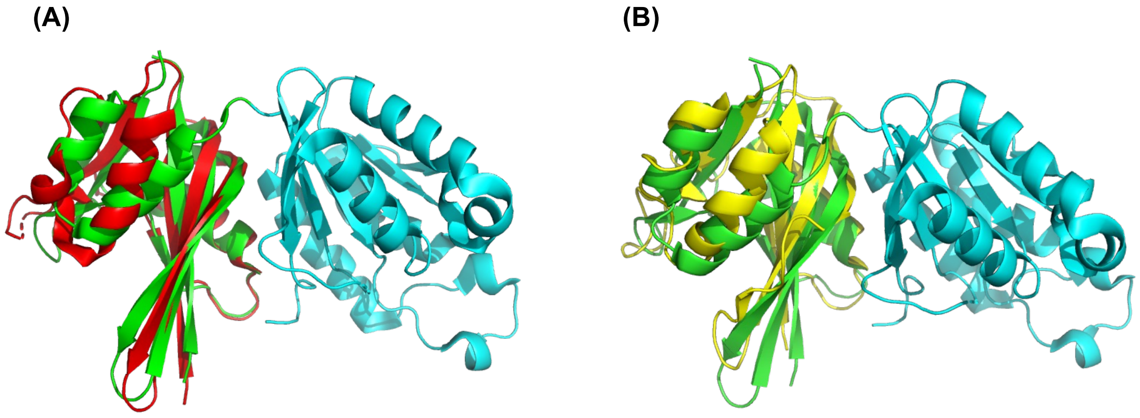

Figure S9: Structural comparison of *Eco*MutT, *Msm*MutT1, and *Msm*MutT2. Comparison of *M. smegmatis* MutT1 (*Msm*MutT1 NTD green colour; PDB entry 5GGB; [22]) with its structural homologues, panels: **(A)** *E. coli* MutT (*Eco*MutT red colour; PDB entry 3A6S;[50]); **(B)** *M. smegmatis* MutT2 (*Msm*MutT2 yellow colour; PDB entry 5ZRG;[51]). The comparison was performed using Edu PyMol software [48].
